# Colorectal Cancer Screening Programs in Latin America: A Systematic Review and Meta-Analysis

**DOI:** 10.1001/jamanetworkopen.2023.54256

**Published:** 2024-02-01

**Authors:** Eleazar E. Montalvan-Sanchez, Dalton A. Norwood, Michael Dougherty, Renato Beas, Maria Guranizo-Ortiz, Miriam Ramirez-Rojas, Douglas R. Morgan, Thomas F. Imperiale

**Affiliations:** 1 Department of Medicine, Indiana University School of Medicine, Indianapolis; 2 Division of Preventive Medicine, The University of Alabama at Birmingham, Birmingham; 3 Division of Gastroenterology and Hepatology, University of North Carolina, Chapel Hill; 4 UNC Rex Digestive Healthcare, Raleigh, North Carolina; 5 Division of Gastroenterology and Hepatology, The University of Alabama at Birmingham; 6 Division of Gastroenterology and Hepatology, Indiana University, Indianapolis; 7 Regenstrief Institute, Indianapolis, Indiana; 8 The Indiana University Simon Cancer Center, Indianapolis

## Abstract

**Importance:**

Colorectal cancer (CRC) is a leading cause of cancer-related mortality globally, with increasing incidence and mortality in Latin America. CRC screening programs can reduce disease burden, but information on screening programs in Latin America is limited.

**Objective:**

To describe characteristics (eg, type of program, uptake, neoplastic yield) of CRC screening programs in Latin America.

**Data Sources:**

PubMed, Ovid MEDLINE, EMBASE, Cochrane, PsycINFO, Web of Science Core Collection, LILACS, and SciELO were searched from inception to February 2023. Relevant references from bibliographies, conference proceedings, and gray literature were considered. The search strategy included English, Spanish, and Portuguese terms.

**Study Selection:**

Included were studies of CRC screening programs in Latin America using fecal immunochemical test (FIT) or colonoscopy as the primary screening method. Four reviewers independently assessed study eligibility based on titles, with review of abstracts and full texts as needed.

**Data Extraction and Synthesis:**

Guidelines from Preferred Reporting Items for Systematic Reviews and Meta-Analyses (PRISMA) were followed for data abstraction and quality assessment. Descriptive information was extracted, and data were pooled using a random-effects model.

**Main outcomes and Measures:**

Program performance indicators included rates of participation and FIT positivity, adenoma detection rate (ADR), advanced adenoma detection rate (AADR), CRC detection rate, and colonoscopy quality indicators.

**Results:**

There were 17 studies included from upper middle-income and high-income countries in Latin America with a total of 123 929 participants. Thirteen studies used FIT as the initial screening method, whereas 4 used screening colonoscopy. The participation rate in FIT-based programs was 85.8% (95% CI, 78.5%-91.4%). FIT positivity rates were 15.2% (95% CI, 9.6%-21.8%) for the 50-ng/mL threshold and 9.7% (95% CI, 6.8%-13.0%) for the 100-ng/mL threshold. For FIT-based studies, the pooled ADR was 39.0% (95% CI, 29.3%-49.2%) and CRC detection rate was 4.9% (95% CI, 2.6%-7.9%); for screening colonoscopy–based studies, the pooled ADR was 19.9% (95% CI, 15.5%-24.8%) and CRC detection rate was 0.4% (95% CI, 0.1%-0.8%).

**Conclusions and Relevance:**

This systematic review and meta-analysis suggests that CRC screening in upper middle-income countries in Latin America is feasible, detecting rates of neoplasia comparable with those of high-income regions. Population-based screening programs should be developed or enhanced in these settings. There is a knowledge gap regarding feasibility and yield of screening programs in lower middle-income countries.

## Key Points

**Question:**

What are the characteristics of colorectal cancer screening programs in Latin America?

**Findings:**

This systematic review and meta-analysis included 17 studies conducted in upper middle-income and high-income countries in Latin America; the pooled participation rate in fecal immunochemical test (FIT)-based programs was 85.8%. For a positive FIT, detection rates were 39.0% for any adenoma, 13.3% for advanced adenomas, and 4.9% for colorectal cancer; these yields for neoplasia were comparable with those observed in high-income settings.

**Meaning:**

These findings suggest that establishing population-level structured screening programs in Latin America, at least in upper middle-income countries, would be effective at reducing colorectal cancer–related disease burden, as they have been in higher-income regions.

## Teaser

This systematic review and meta-analysis characterizes and quantifies colorectal cancer screening program coverage and performance in Latin American countries, with attention to screening test used, program structure, and geographic region.

## Introduction

Colorectal cancer (CRC) is the second-leading cause of cancer-related mortality globally, disproportionately affecting high-income countries.^1,2,3^ CRC incidence and mortality are increasing in low middle-income countries (LMICs) and high middle-income countries (HMICs). This includes Latin America,^2,3,4,5,6,7^ predominately comprised of LMICs and HMICs in economic transition. CRC is the second-most common malignant neoplasm among Latinos in the US.^8^ This upward trend in the Americas is likely due to both westernization of lifestyle and increased life expectancy.^2,9,10,11^ CRC incidence is predicted to increase by 60% in the Americas by 2030.^6,12^

CRC screening has been shown to reduce disease incidence and mortality.^13,14,15^ Globally there are multiple national and professional guidelines recommending screening using a variety of methods starting at different ages,^6,13,16,17,18^ and they generally consist of either 1-step primary colonoscopy or a less-invasive 2-step method of noninvasive testing (with fecal occult blood test or multitarget stool DNA) with colonoscopy for those with a positive noninvasive test. The staged method has the advantages of convenience, ease of scalability, and lower cost, and it may be the preferred method for new CRC screening programs in resource-limited settings.^19^

Studies of CRC screening programs in Latin America are sparce due to the lack of resources for sustainable nationally scaled programs. The first CRC screening program to be established in Latin America was in Uruguay in 2005.^20^ Since then, Brazil, Mexico, and Chile have developed national screening guidelines and focused programs.^21^

Although CRC incidence is increasing,^2,22,23,24,25^ the true incidence of CRC in much of Latin America is still unknown because of the lack of reliable cancer registries.^26,27^ The incidence of precancerous polyps is even less known. To characterize and quantify screening program coverage and performance in Latin American countries, we performed a systematic review and meta-analysis of studies of CRC screening in these countries, with attention to screening test used, program structure, and geographic region.

## Methods

The protocol for this systematic review and meta-analysis was registered in PROSPERO (CRD42022322437) and conducted according to the Preferred Reporting Items for Systematic Reviews and Meta-analyses (PRISMA) reporting guideline.^28^ We systematically searched PubMed, Ovid MEDLINE, EMBASE, and Cochrane from inception to February 2023. Keywords and MeSH terms used are shown in eTable 1 in Supplement 1. To identify studies on CRC screening programs in Latin America, a comprehensive search strategy was developed and applied to 5 other databases: PsycINFO (EBSCO Information Services), Web of Science Core Collection, LILACS (Latin American and Caribbean Health Sciences Literature/Literatura Latino-Americana e do Caribe em Ciências da Saúde), and SciELO (Scientific Electronic Library Online).

We conducted 8 separate searches in Google Scholar to discover additional relevant gray literature. When searching Latin American databases and Google Scholar, we included the terms translated into Spanish and Portuguese. Results from all searches are in eTable 1 in Supplement 1. The searches were developed and executed by a medical librarian (M.R.). The results from all databases were aggregated in Endnote version 20 (Clarivate) and deduplicated using the Covidence tool.

### Study Selection (Inclusion and Exclusion Criteria)

We sought to include CRC screening programs that used either fecal immunochemical test (FIT) or open-access colonoscopy as the principal test. We excluded studies evaluating follow-up colonoscopies (after a prior negative screen), programs using several different screening tests (such as computed tomography, magnetic resonance imaging, and fecal DNA tests), and studies that only looked at population rather than individual patient outcomes. If 2 publications described the same study population or overlapping research protocols, we included the study with the larger sample size.

### Data Extraction

Four independent reviewers (E.M.S., R.B., M.G., D.A.N.) reviewed titles and abstracts, followed by full texts to determine study eligibility. Two independent reviewers extracted descriptive information on author, year of publication, year of conduction of the study, country, World Bank country classification at time of study, study type and design, sample size, funding source, recruitment strategy, type of screening program, and FIT cutoff. We attempted to contact corresponding authors to obtain missing information from their studies. Discrepancies were resolved by consensus and adjudicated (M.K.D., E.M.S., D.R.M., T.I.).

Sampling methods were categorized as voluntary response, convenience sampling, and population-based studies. Voluntary response, also known as self-selection sampling, occurs when individuals or participants respond to a public invitation to enroll in a study. Convenience sampling involves recruiting individuals who are easily accessible and available for the research study, such as a certain clinic panel. Population-based sampling, also known as probability sampling or random sampling, aims to select a sample that represents the entire population accurately.

### Study Quality Assessment

We assessed the risk of bias (ROB) of included studies using the Newcastle-Ottawa Scale version for cross-sectional studies to assess 9 items across 3 domains: (1) selection of the study groups, (2) comparability of groups, and (3) exposure or outcome according to the study design. We assigned 1 point for each item (2 points for a comparability item), for a maximum total score of 9 points.^29^ Scores of 8 to 9 were defined as low ROB, scores of 5 to 7 as medium ROB, and scores of 1 to 4 as high ROB. ROB was assessed independently by 2 reviewers (E.M.S., R.B.), with discrepancies resolved through discussion with a third reviewer (M.K.D.).

### Data Synthesis

Studies were classified by screening modality to be either FIT-based (patients underwent colonoscopy only if their FIT test was positive) or colonoscopy-based (all patients offered colonoscopy, with or without paired FIT). The outcomes were the following: adenoma detection rate (ADR: percentage of colonoscopies with either nonadvanced adenomas, advanced adenomas, or CRC), advanced adenoma detection rate (AADR: percentage of colonoscopies with either advanced adenoma or CRC), CRC detection rate, and colonoscopy quality indicators (cecal intubation rate, mean withdrawal time, and bowel preparation score);^30^ for FIT-based programs, we also assessed the study uptake rate (number of invited individuals who returned the FIT), FIT positivity rate (proportion of individuals who tested positive), and rate of follow-up colonoscopy for positive FITs.

### Statistical Analysis

A random-effects model was used to pool the rates with proportions and 95% CIs for the studies with data available.^31,32^ Freeman-Tukey double arcsine transformation was used to stabilize the variances,^33^ and heterogeneity was assessed by the inconsistency index (*I*^2^). Funnel plots were used to evaluate small-study effects (mainly publication bias), along with the Egger test for statistical significance of funnel plot asymmetry (2-sided *P* < .05 statistically significant). Metaregression random-effects models and subgroup analyses according to a variety of study features (eg, sampling strategy), screening test (eg, FIT hemoglobin cutoff), and patient population (eg, familial risk) factors were used to investigate statistical heterogeneity of the main pooled estimates. All analyses were performed using the metan packages in Stata 18 (StataCorp).

## Results

A total of 5661 articles were identified from the combined databases, with 4267 citations remaining after deduplication. We excluded 3950 based on content of titles and abstracts. We assessed 44 full-text articles for eligibility, and we included 17 in the review with a total of 123 929 participants (eFigure 1 in Supplement 1**)**. Reasons for excluding 27 studies at the full text stage are listed in eTable 2 in Supplement 1.

### Study Characteristics and Quality Assessment

All included studies were published after 2006, with study data collection occurring from 1999 to 2019. All studies were conducted in HMICs or high-income countries in Latin America, defined by World Bank classification at the start date of the specific study. Eleven (65%) of 17 were conducted in South America (Argentina [n = 2]; Brazil [n = 4]; Chile [n = 3]; Uruguay [n = 2]),^20,34,35,36,37,38,39,40,41,42,43^ and the remaining 6 studies were conducted in Mexico and Central America (Mexico [n = 4]; Costa Rica [n = 1]; Panama [n = 1]).^44,45,46,47,48,49^ The articles were published in English (n = 10),^20,34,37,40,42,43,44,47,48,49^ Spanish (n = 6),^35,36,38,39,45,46^ and Portuguese (n = 1).^41^ Funding sources were governmental (n = 5), governmental with other agencies (n = 2), private (n = 3), international (n = 2), and not reported (n = 5). Four studies were colonoscopy-based without prior FIT.^34,36,44,49^ Of the 13 FIT-based studies, several included a cohort of high-risk study participants (familial/genetic risk or symptomatic)^38,40,41,42^ who underwent colonoscopy; for our analysis, however, we only included the subset with a positive FIT.^20,40^ Most FIT studies reported hemoglobin cutoffs of 50 ng/mL (5 studies) or 100 ng/mL (6 studies), although 2 did not report the cutoff.^41,46^ Descriptive characteristics of included studies are presented in Table 1.

**Table 1.** Descriptive Characteristics of Included Studies

Study (country)	Project period	Language	Income level (WBI)	Sample size	Type of screening program	Funding	Risk of bias^a^	FIT brand	FIT cutoff value, ng/mL
Fenocchi et al,^20^ 2006 (Uruguay)	1999-2004	English	UMICs	11734	FIT	International (JICA)	Low	OC-Hemodia	100
Rettally,^49^ 2008 (Panama)	2004-2007	English	HICs	306	Colonoscopy	NR	Medium	NA	NA
Silva et al,^36^ 2011 (Chile)	2003-2008	Spanish	UMICs	1158	Colonoscopy	Private	Medium	NA	NA
Lopez-Kostner et al,^38^ 2012 (Chile)	2007-2009	Spanish	UMICs	6348	FIT	Governmental	Low	OC-sensor Micro, Eiken	100
Fenocchi et al,^40^ 2015 (Uruguay)	2015	English	UMICs	902	FIT	NR	Low	OC-Eiken	100
Garcia-Osogobio et al,^44^ 2015 (Mexico)	2009-2010	English	UMICs	123	Colonoscopy	Private	Low	NA	NA
Braga et al,^41^ 2017 (Brazil)	2014-2015	Portuguese	UMICs	438	FIT	NR	Medium	NR	NA
Teixeira et al,^37^ 2017 (Brazil)	2015-2016	English	UMICs	1039	FIT	Private	Medium	OC-Light	50^b^
Alfaro-Seguro et al,^46^ 2020 (Costa Rica)	2017-2019	Spanish	UMICs	53003	FIT	Governmental	Low	NR	NA
Galvez-Rios et al,^45^ 2020 (Mexico)	2015-2016	Spanish	UMICs	900	FIT	Governmental	Medium	OC FIT-CHEK	100
Manzano-Robleda et al,^47^ 2020 (Mexico)	2017-2019	English	UMICs	810	FIT	Governmental/nonprofit	Low	OC FIT-CHEK	50^c^
Okada et al,^42^ 2016 (Chile)	2012-2014	English	UMICs	26444	FIT	Governmental	Low	NR	100
Remes-Troche et al, 48 2020 (Mexico)	2015-2016	English	UMICs	473	FIT	International (US NCI)	Medium	OC FIT-CHEK	100
Averbach et al,^34^ 2021 (Brazil)^d^	2014-2017	English	UMICs	2022	Colonoscopy	Governmental/private	Low	Bioptix	NA
Fernandez et al,^39^ 2021 (Argentina)	2019-2020	Spanish	UMICs	730	FIT	Governmental	Medium	SD. BIOLINE FOB	50^b^
Guimaraes et al,^43^ 2021 (Brazil)	2015-2017	English	UMICs	6737	FIT	NR	Low	Hemosure	50^b^

Abbreviations: FIT, fecal immunochemical test; HICs, high-income countries; JICA, Japan International Cooperation Agency; NA, not available; UMICs, upper- and middle-income countries; US NCI, United States National Cancer Institute; WBI, World Bank Index.

^a^ Per dual review using the Newcastle-Ottawa Scale for comparative studies (Wells et al,^29^ 2014).

^b^ Values converted from ug/g to ng/mL.

^c^ The 50 ng/mL cutoff was used for comparability with the other studies; results were also provided for cutoffs of 20 ng/mL and 100 ng/mL.

^d^ Averbach et al administered colonoscopies and FIT to all individuals.

All but 1 study had an eligibility age of 50 years or older.^44^ Most studies excluded individuals at elevated risk, such as those with a personal or family history of colorectal neoplasm or inflammatory bowel disease. Where studies included a group of diagnostic or nonaverage risk screening procedures, we included only the average-risk screening population in the analyses whenever possible,^49^ although some studies still had some contamination of familial risk^36,42,48,49^ or symptomatic participants,^41^ which led to lower comparability ratings in risk-of-bias assessment. All studies were judged to have a low or medium risk of bias according to Newcastle-Ottawa (eTable 3 in Supplement 1). Recruitment strategies included media advertisement only (n = 5), personal invitation (n = 7), combination of personal and media (n = 3), and unspecified (n = 2). Strategies for sampling and recruiting are presented in eTable 4 in Supplement 1.

### FIT-Based Programs: Participation

**Figure 1.**
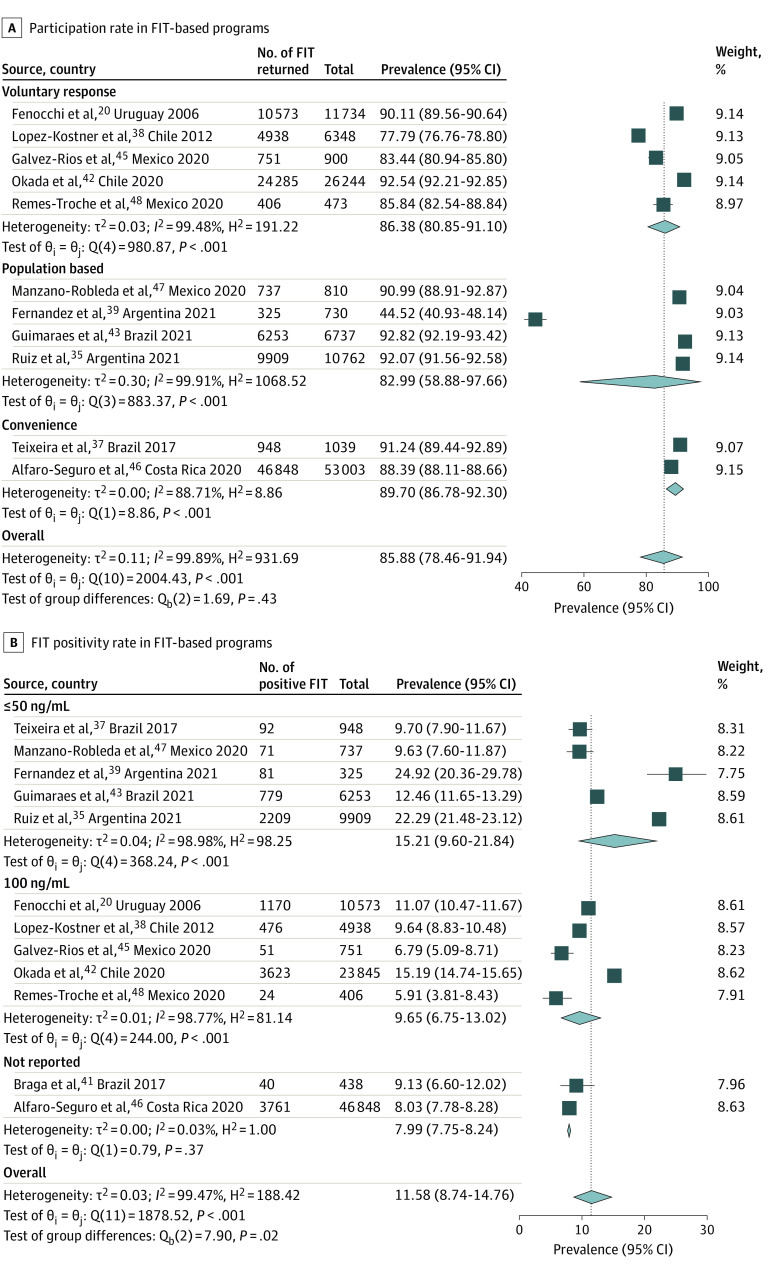
Forest Plots of the Outcomes of Colorectal Cancer Screening Programs in Latin America FIT indicates fecal immunochemical test.

A summary of pooled estimates of clinical outcomes and performance indicators is presented in Table 2. FIT studies reported participation rates ranging from 44.5% to 92.8% among 118 780 individuals invited, with an overall pooled participation rate of 85.8% (95% CI, 78.5%-91.4%; *I*^2^ = 99.9%). Uptake was not associated with sampling strategy (voluntary response: 86.4% [95% CI, 80.9%-91.1%]; convenience: 89.7% [95% CI, 86.8%-92.3%]; population-based: 83.0% [95% CI, 58.9%-97.7%]; *P* = .43) (Figure 1A) or recruitment strategy (media recruitment: 87.9% [95% CI, 84.2%-91.1%]; individual recruitment: 88.9% [95% CI, 83.5%-93.4%]; *P* = .72) (eFigure 3 in Supplement 1). The pooled participation rate was 85.0% (95% CI, 72.5%-94.2%) in South America and was 87.4% (95% CI, 84.0%-90.4%) in Mexico and Central America (*P* = .68) (eFigure 2 in Supplement 1). Studies with larger sample sizes reported higher participation rates (sample size <1000: 78.0% [95% CI, 56.2%-93.8%]; sample size 1000-10 000: 88.0% [95% CI, 77.9%-95.3%]; sample size >10 000: 90.2% [95% CI, 88.0%-92.2%]), although this was not a statistically significant difference (*P* = .33) (eFigure 4 in Supplement 1).

**Table 2.** Pooled Estimates of Performance Indicators for FIT and Colonoscopy-Based Programs

Variable	Studies, No.	Population, No.	Positive test, No.	Range, %	Point estimate, % (95% CI)^a^
Participation rate					
Overall	12	118 780	105 971	44.5-92.8	85.8 (78.5-91.4)
Voluntary	5	45 699	40 513	77.8-98.2	86.4 (80.9-91.1)
Population-based	4	19 039	17 224	44.5-92.82	83 (58.9-97.7)
Convenience	3	54 042	48 234	88.4-91.24	89.7 (86.8-92.3)
FIT positivity rate					
Overall	12	105 971	12 377	5.9-24.9	11.6 (8.7-14.8)
50 ng/dL	5	18 172	3232	9.6-24.9	15.2 (9.6-21.8)
100 ng/dL	5	40 513	5344	5.9-15.2	9.7 (6.8-13.0)
Not reported	2	47 286	3801	8.0-9.1	8 (7.8-8.2)
Detection rates per positive FIT^b^					
ADR	12	12 377	3377	5.5-55.07	28.5 (19.1-38.8)
AADR	8	7788	282	1.1-28.2	8.3 (3.5-14.9)
CRC detection rate	12	12 377	410	0.0-12.0	3.5 (1.6-6.0)
Any adenoma (ADR)					
FIT-positive^c^	12	9373	3377	13.9-65.0	39 (29.3-49.2)
Screening colonoscopy	4	3494	699	16.6-26.1	19.9 (15.5-24.8)
Advance Adenoma (AADR)					
FIT-positive^c^	10	7240	648	2.2-34.5	13.3 (7.4-20.5)
Screening colonoscopy	2	2258	62	2.6-3.9	2.9 (1.8-4.3)
CRC detection rate					
FIT-positive^c^	12	9373	420	0.0-15.1	5.4 (3.1-8.2)
Screening colonoscopy	4	3494	15	0.0-1.3	0.4 (0.1-0.8)
Colonoscopy quality indicators					
Cecal intubation rate	12	12 203	11 515	86.8-100.0	95.7 (92.7-97.9)
Withdrawal time, mean, min	4	4825	9.9	7.3-17.0	8.4 (4.3-12.4)
Bowel preparation (adequate)	4	6459	6109	83.5-99.6	94.6 (86.6-99.5)

Abbreviations: AADR, advanced adenoma detection rate; ADR, adenoma detection rate (any adenoma); CRC, colorectal cancer; FIT, fecal immunochemical test.

^a^ Random-effects meta-analysis of proportions.

^b^ In FIT-based programs.

^c^ Rates per colonoscopy after positive FIT, from FIT-based programs only.

### FIT-Based Programs: Positivity Rate and Outcomes

Twelve FIT studies reported FIT positivity rates, which ranged from 5.9% to 24.9%, with a pooled FIT positivity rate of 11.6% (95% CI, 8.7%-14.8%; *I*^2^ = 99.5%). This rate was significantly associated with FIT threshold, with 15.2% (95% CI, 9.6%-21.8%) positivity for thresholds of 50 ng/dL or less and 9.7% (95% CI, 6.8%-13.0%) for 100 ng/dL (*P* = .02) (Figure 1B). Approximately three-fourths (79.6% [95% CI, 65.4%-90.8%]) of individuals with a positive FIT underwent diagnostic colonoscopy (eFigure 5 in Supplement 1). There was minimal description of interventions to navigate FIT-positive participants to colonoscopy beyond efforts of study personnel, although the 2 underperforming outlier studies were more pragmatic^35,39^ pilot studies that acknowledged limitations of the follow-up colonoscopy process during the study period.

The pooled ADR per positive FIT test was 28.5% (95% CI, 19.1%-38.8%); pooled AADR per positive FIT test was 8.3% (95% CI, 3.5%-14.9%); and pooled CRC detection rate per positive FIT test was 3.5% (95% CI, 1.6%-6.0%) (eFigures 5-7 in Supplement 1). These outcomes provide the neoplasia yield of the entire FIT-based program, accounting for the variation in colonoscopy follow-up. There was no difference in ADR, AADR, or CRC detection rate by FIT cutoff. ADR was 29.4% (95% CI, 9.1%-55.4%) for 50 ng/dL and 29.9% (95% CI, 24.2%-36.0%) for 100 ng/dL (*P* = .97) (eFigure 6 in Supplement 1); AADR was 9.2% (95% CI, 2.1.8%-20.7%) for 50 ng/dL and 11.9% (95% CI, 7.8%-16.5%) for 100 ng/dL (*P* = .02) (eFigure 7 in Supplement 1); and CRC detection rate was 4.7% (95% CI, 1.3%-9.8%) for 50 ng/dL and 2.9% (95% CI, 0.5%-6.8%) for 100 ng/dL (*P* = .86) (eFigure 8 in Supplement 1).

### Yield of Colonoscopy

**Figure 2.**
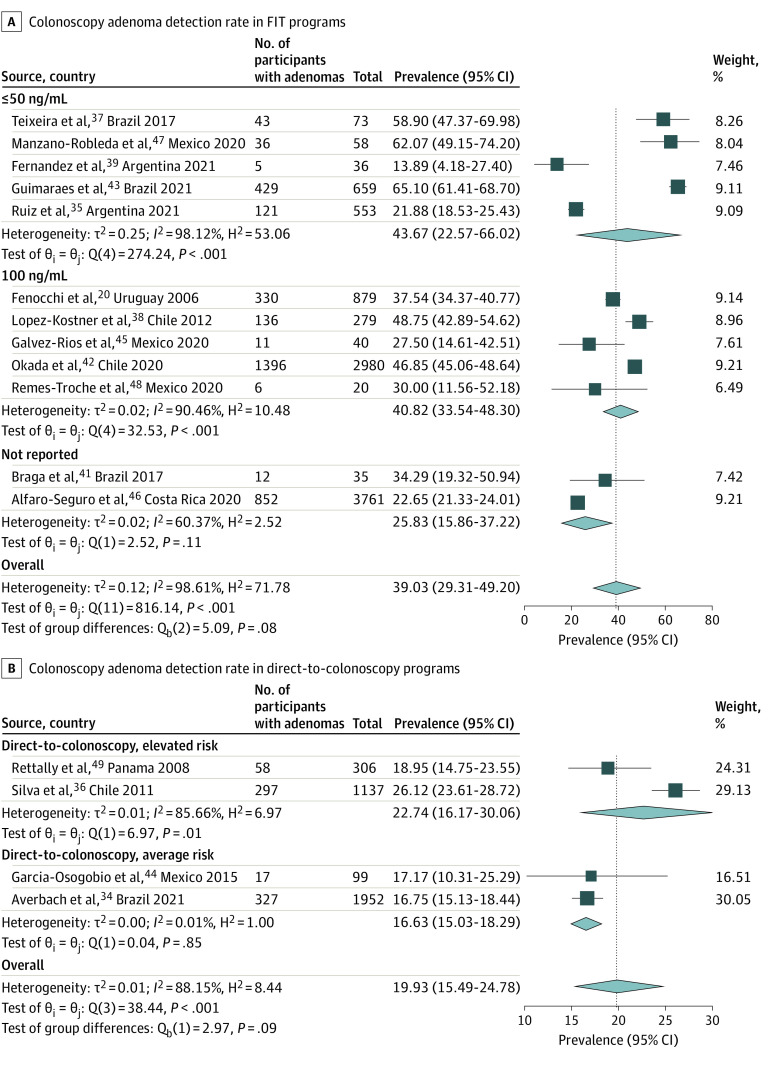
Colonoscopy Adenoma Detection Rate in FIT and Direct-to-Colonoscopy Programs FIT indicates fecal immunochemical test.

**Figure 3.**
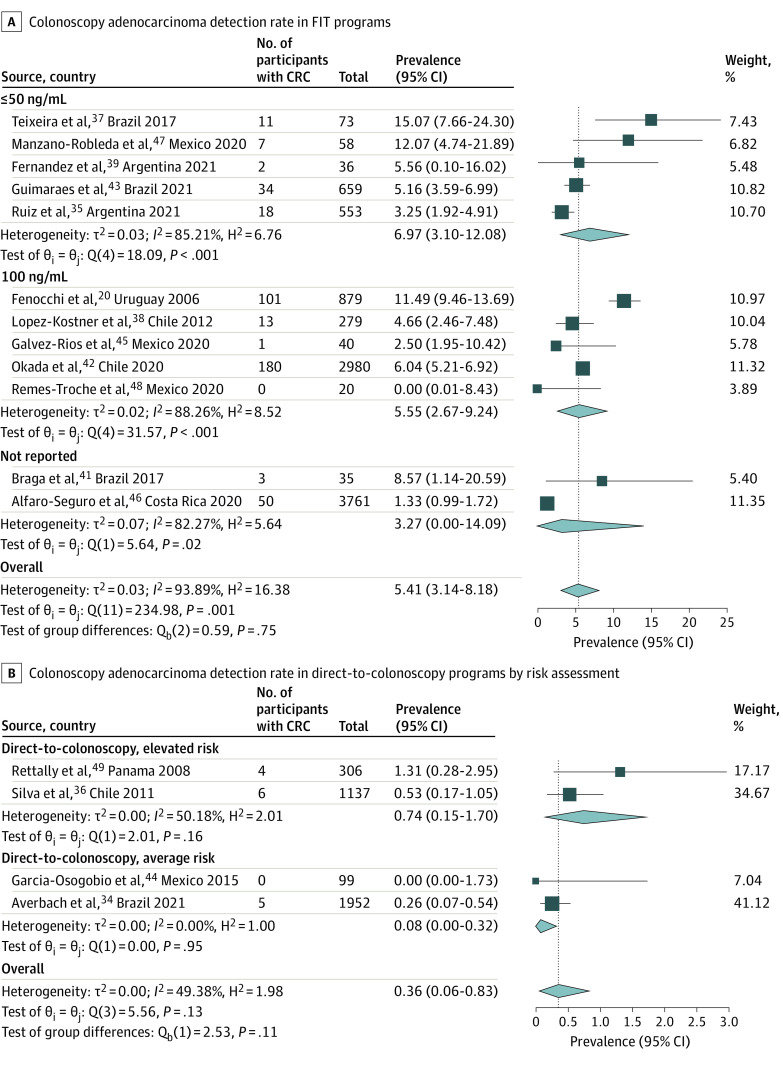
Colonoscopy Adenocarcinoma Detection Rate in FIT and Direct-to-Colonoscopy Programs CRC indicates colorectal cancer; FIT, fecal immunochemical test.

A total of 18 037 colonoscopies were performed among all studies. ADR per procedure ranged from 13.9% to 65.1%, higher following a positive FIT (39.0% [95% CI, 29.3%-49.2%]) (Figure 2A) than for primary screening colonoscopy (19.9% [95% CI, 15.5%-24.8%]) (Figure 2B**)**. AADR data were available in 12 studies (10 FIT programs and 2 screening colonoscopy programs), with a pooled rate of 13.3% (95% CI, 7.4%-20.6%) for FIT (eFigure 9 in Supplement 1) and 2.9% (95% CI, 1.8%-4.3%) for primary screening colonoscopy (eFigure 10 in Supplement 1). The CRC detection rate ranged from 0% to 15.1%, with a pooled rate of 3.5% (95% CI, 1.6%-6.0%) (eFigure 11 in Supplement 1). CRC detection was higher in FIT-based programs (5.4% [95% CI, 3.1%-8.2%]) (Figure 3A) compared with primary screening colonoscopy (0.4% [95% CI, 0.1%-0.8%]) (Figure 3B), a statistically significant difference (*P* < .001) (eFigure 11 in Supplement 1).

### Colonoscopy Quality

A total of 12 studies reported at least 1 additional quality indicator for colonoscopy beyond ADR. Cecal intubation rate was reported in 12 studies, with a pooled rate of 95.7% (95% CI, 92.7%-98.0%) (eFigure 12 in Supplement 1). Mean withdrawal time from the cecum was reported in 4 studies, with a mean time of 8.4 minutes (95% CI, 4.3-12.4 minutes) (eFigure 13 in Supplement 1) and rate of adequate bowel preparation in 4 studies with a pooled estimate of 94.6% (95% CI, 86.6%-99.5%) (eFigure 14 in Supplement 1). Overall, only 5 studies reported the type of colonoscopy equipment utilized, which were comparable (Olympus 180 or Fujifilm 530, each with high-definition endoscopes), except for Fenocchi et al,^20^ which used Olympus 140-series.^20^ One study used endoscopic accessories,^47^ resulting in one of the highest ADRs. Another study with high ADR used indigo carmine chromoendoscopy in all procedures, with a correspondingly longer withdrawal time.^43^

### Heterogeneity and Subgroup Analyses

Assessments for heterogeneity (Galbraith plots) and publication bias (Funnel plot of small study effects with Egger *P* value) can be found in eFigures 14, 15, 16, 17, and 18 in Supplement 1. No significant small study effects were detected, but statistical heterogeneity was high in all analyses. We explored this heterogeneity in the FIT-based studies for the principal outcomes of neoplasia yield (detection rates of adenomas, advanced adenomas, and CRC) with metaregression, which was a single variable regression given the limited number of studies (eTable 5 in Supplement 1). Among significant modifiers, populations with more female individuals demonstrated higher ADRs and AADRs; greater mean age was associated with higher rates of CRC. Exclusion of studies using visualization enhancements for polyp detection decreased the overall ADR from 39.0% (95% CI, 29.3%-49.2%) to 34.2% (95% CI, 25.5%-43.3%) (eFigure 19 in Supplement 1), but also realigned the expected direction of ADR as higher at higher FIT cutoff (40.8% [95% CI, 33.5%-48.3%] at 100 ng/mL vs 30.4% [95% CI, 8.3%-58.7%] at 50 ng/mL; eFigure 20 in Supplement 1). Stratification by familial risk was not associated with detection of CRC, however, at least among FIT positives (eFigure 21 in Supplement 1).

To explore the effect of such outliers on summary estimates, in addition to the Galbraith and forest plots, we performed leave-one-out meta-analyses for neoplasia outcomes per colonoscopy in FIT-based studies (eFigures 22-24 in Supplement 1), although no single study affected overall ADR more than 2.5%, AADR more than 2.0%, or CRC detection rate more than 0.7%. Overall, while these subgroup analyses helped to explain much of the clinical heterogeneity, the *I*^2^ metric remained high in all main analyses.

## Discussion

To our knowledge, this is the first systematic review to quantitatively evaluate the nascent CRC screening programs in Latin America. This review found that colon cancer screening, either with a FIT-based or screening colonoscopy–based strategy, was successfully implemented in a variety of upper middle-income countries throughout Latin America, with high yields of neoplasia. The detection rates of adenomas (19.9% for screening colonoscopy and 39.0% after positive FIT), advanced adenomas (2.9% for screening colonoscopy and 13.3% after positive FIT), and CRC (0.4% for screening colonoscopy and 5.4% after positive FIT) were comparable to those in mature programs in North American and European countries, where ADR averages 23% to 42% and CRC detection 0.5% in direct colonoscopy,^50,51,52,53,54^ and ADR is 48% and CRC detection is 5.1% among individuals with a positive FIT.^55^ Other program metrics are also in the same general range (FIT positivity: 5.9%-24.9% vs 4.3%-10.1%) or slightly superior (participation rate: 44.5%-92.8% vs 48.2%-64.7%) to those of higher-income regions.^7,25,56^ Population-based screening programs may represent a cost-efficient opportunity to address the growing burden of CRC in Latin America, with the caveat that this review did not identify studies from LMICs, nor did it identify screening programs that were unsuccessful.

Limited resources, infrastructure, and lack of public awareness have posed challenges for population-based CRC screening programs in Latin America.^6^ Despite these obstacles, our review of upper-middle-income countries found high uptake (85.8%) of screening measures, with FIT-based studies reporting greater than 75% colonoscopy completion rates after positive FIT. Data on quality metrics, such as cecal intubation rates (95.7%) and bowel preparation (94.6%), was limited, however, it aligns with global standards, suggesting the feasibility of CRC screening in Latin American upper-middle-income countries.^30,57,58,59,60^

The rising incidence and mortality of CRC in Latin American countries underscores the need for screening programs at the population level. Although our study found that both primary colonoscopy and FIT-based programs were associated with higher detection rates, given the resource constraints in these countries, a stool-based strategy is likely most feasible and cost-effective.^61,62,63,64,65,66,67^ A stool-based strategy allows for country-specific FIT cutoffs tailored to CRC prevalence and colonoscopy capacity.^68,69^ Stool test distribution is more easily integrated into existing public health infrastructure, such as population-based *H. pylori*, cervical cancer, or diabetes screening.

Regardless of a country’s initial screening strategy, colonoscopy resources are a crucial and potentially rate-limiting factor. Developing these resources may be guided through international collaboration, as previously modeled in Latin America with Japanese support of the Chilean CRC national program.^38,42^ Established in 2012, this collaboration between a private entity, the government, and an academic institution demonstrated promising results,^42^ with significant improvement in ADR and cancer detection rate after local Chilean physicians received colonoscopy skills–improvement training.^42^ In addition to overseas collaborations, however, our study suggests that enough Latin American centers have developed sufficient expertise in CRC screening that intracontinental collaborations could fill some of this need for mentorship and professional development for the more nascent programs. Furthermore, regional networks among Latin American countries, with potential advocacy from the Pan-American Health Organization (PAHO), to share screening outcomes will strengthen knowledge of both disease epidemiology and optimal screening practices. Additionally, national health ministries’ commitment to program funding will be important for sustainability, even if research or nongovernmental funding is used initially.^70^

### Limitations

This systematic review and meta-analysis had limitations. The main limitation of this review is statistical heterogeneity among the included studies. This is due to a variety of causes, both clinical and statistical. In the pooled analysis of participation rate for FIT-based studies, all studies except 1 (Fernandez et al^39^) had participation rates from 78% to 93%, and 8 of 10 studies fell between 83% and 93%. This variation may not be considered clinically important, but even with exclusion of the most extreme outlier (Fernandez et al^39^)—a small study with shortcomings in project implementation—*I*^2^ remains high at 99.5%. There are other study factors, such as recruitment and sampling methods, that likely contribute to this variation. However, the majority of this heterogeneity is purely statistical, due to the number of large studies with low within-study variance, which artificially inflates the *I*^2^ in the presence of relatively small between-study variances.

Clinical heterogeneity is evident in FIT positivity and ADR rates. Two Argentine pilot programs, being new, involved nonresearch staff, potentially leading to errors in FIT interpretation and inclusion of symptomatic patients. While ADR outliers could be linked to equipment differences, we could not identify other factors that may have contributed to heterogeneity. Stratification by (familial) risk or FIT positivity threshold surprisingly did not significantly affect neoplasia yields, possibly due to unreported factors or true population differences.^20,37^ Incomplete reporting of FIT cutoff by studies and the ecological nature of comparisons limit the ability to explore sources of heterogeneity.^41,46^ Caution is advised in interpreting summary estimates, serving as the best available estimates for future study planning based on a limited and emerging literature.

Teixeira et al’s study^37^ reported high neoplasia detection despite normal FIT positivity in an average-risk population. Possible explanations include unreported endoscopic accessory use or high-detecting endoscopists. Another outlier study from Uruguay suggested a higher-risk population with disproportionate AADR and CRC detection due to lower image resolution colonoscopes.^20^ The main negative outlier for ADR also showed low FIT positivity and participation rates, with ongoing protocol development and potential errors in FIT interpretation and participant recruitment acknowledged by the authors.^39^

Other limitations of this review highlight future challenges in stemming the impending wave of CRC in Latin America. This review highlights challenges in addressing the growing CRC burden in Latin America. Two countries (Costa Rica, Panama) lacked reported CRC screening programs in the 2016 PAHO report, and we identified studies from 7 countries, none of which were LMICs. While no publication bias was found in our funnel plots, there could be unpublished CRC screening efforts due to implementation failures, constituting infeasibility. The LMIC “double cancer burden” is well-documented,^26,71^ with increasing rates of CRC in Latin American lower- and upper-middle-income countries, emphasizing the need for research on CRC prevention in the region.^3,71^

## Conclusions

This systematic review and meta-analysis found both the substantial burden of colorectal neoplasia and the feasibility of organized screening programs in Latin America. It also highlights the need for more data on CRC burden or screening feasibility in the Latin American LMICs. CRC incidence is rising in Latin America as fast as anywhere in the world, warranting effective preventive measures, particularly with cost-effective, FIT-based screening programs. CRC screening should become a greater research and public health priority in Latin America.

## Notes

Supplement 1. **eTable 1.** Search Strategy **eFigure 1.** Flow Diagram of Study Inclusion **eTable 2.** Excluded Studies and Reasons for Exclusion **eTable 3.** Risk of Bias Using the New Castle Ottawa Scale for Comparative Studies **eTable 4.** Strategies for Sampling and Recruitment of Included Studies **eTable 5.** Univariate Meta Regression **eFigure 2.** Participation Rate (FIT Given/FIT Returned) by Region **eFigure 3.** Participation Rate (FIT Given/FIT Returned) by Recruitment Strategy **eFigure 4.** Participation Rate (FIT Given/FIT Returned) by Sample Size **eFigure 5.** Colonoscopy Follow-Up (Colonoscopies Performed After a Positive FIT Test) **eFigure 6.** Outcome ADR (Adenoma Detection Rate) per Positive FIT Test in FIT Based Programs **eFigure 7.** Outcome AADR (Advanced Adenomas Detection Rate) per Positive FIT Test in FIT Based Programs **eFigure 8.** Outcome CRCDR (Colorectal Cancer Detection Rate) per Positive FIT Test in FIT Based Programs **eFigure 9.** Yield of Colonoscopy: AADR (Advanced Adenomas Detection Rate). FIT Programs **eFigure 10.** Yield of Colonoscopy: AADR (Advanced Adenomas Detection Rate). Direct-to-Colonoscopy Programs **eFigure 11.** Yield of Colonoscopy: CRC-DR (Adenocarcinoma Detection Rate) by Type of Program **eFigure 12.** Colonoscopy Quality Indicators: Cecal Intubation Rate **eFigure 13.** Colonoscopy Quality Indicators: Mean Withdrawal Time From Cecum (Mean, Minutes) **eFigure 14.** Colonoscopy Quality Indicators: Adequate Bowel Preparation **eFigure 15.** Heterogeneity and Publication Bias (Small Study Effects) for FIT Based Studies **eFigure 16.** Heterogeneity and Publication Bias (Small Study Effects) for Outcomes per Positive FIT Test in FIT Based Programs **eFigure 17.** Heterogeneity and Publication Bias (Small Study Effects) for Outcomes in Direct-to-Colonoscopy Programs **eFigure 18.** Heterogeneity and Publication Bias (Small Study Effects) for Colonoscopy Quality Indicators **eFigure 19.** ADR (Adenomas Detection Rate) FIT Programs by Risk: Leave-One-Out Analysis **eFigure 20.** ADR (Adenomas Detection Rate) FIT Programs by FIT Cut-Off: Leave-One-Out Analysis **eFigure 21.** CRC (Adenocarcinoma Detection Rate) FIT Programs: Leave-One-Out Analysis **eFigure 22.** ADR (Adenomas Detection Rate) FIT Programs: Leave-One-Out Analysis **eFigure 23.** AADR (Advanced Adenomas Detection Rate) FIT Programs: Leave-One-Out Analysis **eFigure 24.** CRC (Adenocarcinoma Detection Rate) FIT Programs: Leave-One-Out Analysis

Supplement 2.
Data Sharing Statement
