# Supplementary material for: Colorectal Cancer Screening Programs in Latin America: A Systematic Review and Meta-Analysis
Source: JAMA Netw Open. 2024 Feb 1;7(2):e2354256. doi: 10.1001/jamanetworkopen.2023.54256 (PMC10835514; doi:10.1001/jamanetworkopen.2023.54256)
Supplement: Supplement 1. — eTable 1. Search Strategy eFigure 1. Flow Diagram of Study Inclusion eTable 2. Excluded Studies and Reasons for Exclusion eTable 3. Risk of Bias Using the New Castle Ottawa Scale for Comparative Studies eTable 4. Strategies for Sampling and Recruitment of Included Studies eTable 5. Univariate Meta Regression eFigure 2. Participation Rate (FIT Given/FIT Returned) by Region eFigure 3. Participation Rate (FIT Given/FIT Returned) by Recruitment Strategy eFigure 4. Participation Rate (FIT Given/FIT Returned) by Sample Size eFigure 5. Colonoscopy Follow-Up (Colonoscopies Performed After a Positive FIT Test) eFigure 6. Outcome ADR (Adenoma Detection Rate) per Positive FIT Test in FIT Based Programs eFigure 7. Outcome AADR (Advanced Adenomas Detection Rate) per Positive FIT Test in FIT Based Programs eFigure 8. Outcome CRCDR (Colorectal Cancer Detection Rate) per Positive FIT Test in FIT Based Programs eFigure 9. Yield of Colonoscopy: AADR (Advanced Adenomas Detection Rate). FIT Programs eFigure 10. Yield of Colonoscopy: AADR (Advanced Adenomas Detection Rate). Direct-to-Colonoscopy Programs eFigure 11. Yield of Colonoscopy: CRC-DR (Adenocarcinoma Detection Rate) by Type of Program eFigure 12. Colonoscopy Quality Indicators: Cecal Intubation Rate eFigure 13. Colonoscopy Quality Indicators: Mean Withdrawal Time From Cecum (Mean, Minutes) eFigure 14. Colonoscopy Quality Indicators: Adequate Bowel Preparation eFigure 15. Heterogeneity and Publication Bias (Small Study Effects) for FIT Based Studies eFigure 16. Heterogeneity and Publication Bias (Small Study Effects) for Outcomes per Positive FIT Test in FIT Based Programs eFigure 17. Heterogeneity and Publication Bias (Small Study Effects) for Outcomes in Direct-to-Colonoscopy Programs eFigure 18. Heterogeneity and Publication Bias (Small Study Effects) for Colonoscopy Quality Indicators eFigure 19. ADR (Adenomas Detection Rate) FIT Programs by Risk: Leave-One-Out Analysis eFigure 20. ADR (Adenomas Detection Rate) FIT Programs [file jamanetwopen-e2354256-s001.pdf]

# Supplemental Online Content

Montalvan-Sanchez EE, Norwood DA, Dougherty M, et al. Colorectal Cancer Screening Programs in Latin America. *JAMA Netw Open.* 2024;7(2):e2354256. doi:10.1001/jamanetworkopen.2023.54256

**eTable 1.** Search Strategy

**eFigure 1.** Flow Diagram of Study Inclusion

**eTable 2.** Excluded Studies and Reasons for Exclusion

**eTable 3.** Risk of Bias Using the New Castle Ottawa Scale for Comparative Studies

**eTable 4.** Strategies for Sampling and Recruitment of Included Studies

**eTable 5.** Univariate Meta Regression

**eFigure 2.** Participation Rate (FIT Given/FIT Returned) by Region

**eFigure 3.** Participation Rate (FIT Given/FIT Returned) by Recruitment Strategy

**eFigure 4.** Participation Rate (FIT Given/FIT Returned) by Sample Size

**eFigure 5.** Colonoscopy Follow-Up (Colonoscopies Performed After a Positive FIT Test)

**eFigure 6.** Outcome ADR (Adenoma Detection Rate) Per Positive FIT Test in FIT Based Programs

**eFigure 7.** Outcome AADR (Advanced Adenomas Detection Rate) Per Positive FIT Test in FIT Based Programs

**eFigure 8.** Outcome CRCDR (Colorectal Cancer Detection Rate) Per Positive FIT Test in FIT Based Programs

**eFigure 9.** Yield of Colonoscopy: AADR (Advanced Adenomas Detection Rate). FIT Programs

**eFigure 10.** Yield of Colonoscopy: AADR (Advanced Adenomas Detection Rate). Direct-to-Colonoscopy Programs

**eFigure 11.** Yield of Colonoscopy: CRC-DR (Adenocarcinoma Detection Rate) by Type of Program

**eFigure 12.** Colonoscopy Quality Indicators: Cecal Intubation Rate

**eFigure 13.** Colonoscopy Quality Indicators: Mean Withdrawal Time from cecum (mean, minutes)

**eFigure 14.** Colonoscopy Quality Indicators: Adequate Bowel Preparation

**eFigure 15.** Heterogeneity and Publication bias (small study effects) for FIT Based Studies

**eFigure 16.** Heterogeneity and Publication Bias (Small Study Effects) for Outcomes Per Positive FIT Test in FIT Based Programs

© 2024 Montalvan-Sanchez EE et al. *JAMA Network Open.*

**eFigure 17.** Heterogeneity and Publication Bias (Small Study Effects) for Outcomes in Direct-to-Colonoscopy Programs

**eFigure 18.** Heterogeneity and Publication Bias (Small Study Effects) for Colonoscopy Quality Indicators

**eFigure 19.** ADR (Adenomas Detection Rate) FIT Programs by Risk: Leave-One-Out Analysis

**eFigure 20.** ADR (Adenomas Detection Rate) FIT Programs by FIT Cut-Off: Leave-One-Out Analysis

**eFigure 21.** CRC (Adenocarcinoma Detection Rate) FIT Programs: Leave-One-Out Analysis

**eFigure 22.** ADR (Adenomas Detection Rate) FIT Programs: Leave-One-Out Analysis

**eFigure 23.** AADR (Advanced Adenomas Detection Rate) FIT programs: Leave-One-Out Analysis

**eFigure 24.** CRC (Adenocarcinoma Detection Rate) FIT Programs: Leave-One-Out Analysis

This supplemental material has been provided by the authors to give readers additional information about their work.

© 2024 Montalvan-Sanchez EE et al. *JAMA Network Open.*

## eTable1. Search Strategy

| Database | Search Strategy | Result |
| --- | --- | --- |
| Medline (OVID) | 1 exp colonoscopy/<br>2 "Colonography, Computed Tomographic"/<br>3 "occult blood"/<br>4 colonoscop*.ti,ab,kf.<br>5 colonograph*.ti,ab,kf.<br>6 sigmoidoscop*.ti,ab,kf.<br>7 ((colon or colorectal or colo-rectal or colonic or anal or anus or bowel or rectocolonic* or rectal or rectum or sigmoid*) adj3 (endoscop* or tomograph*)).ti,ab,kf.<br>8 ((occult or hidden) adj3 (blood or bleeding or haemorrhag* or hemorrhag*)).ti,ab,kf.<br>9 ((stool or fecal or faecal or faeces or feces) adj3 blood).ti,ab,kf.<br>10 hemoccult.ti,ab,kf.<br>11 (FOB or FOBT* or gFOBT* or iFOBT*).ti,ab,kf.<br>12 ((stool or fecal or faecal or faeces or feces) adj5 (immunologic* or immunochem*)).ti,ab,kf.<br>13 ((stool or guaiac or guajac or guaiacum) adj5 (test or sample*)).ti,ab,kf.<br>14 (((stool or fecal or faecal or faeces or feces) adj3 (DNA or sDNA)) or "sDNA-FIT").ti,ab,kf.<br>15 or/1-14<br>16 exp Colorectal Neoplasms/<br>17 Colonic Polyps/<br>18 exp Colonic Diseases/<br>19 ((colon or colorectal or colo-rectal or colonic or anal or anus or bowel or rectocolonic* or rectal or rectum or sigmoid) adj5 (cancer* or carcinogen* or neoplasm* or neoplasia or neoplastic or adenoma* or adenocarcinoma* or polyp or polyps or carcinoma* or malignan* or tumor* or tumour* or precancer* or premalignan*)).ti,ab,kf.<br>20 or/16-19<br>21 Mass Screening/<br>22 Early Detection of Cancer/<br>23 Early Diagnosis/<br>24 (screening* or screened or screens).ti,ab,kf.<br>25 ((early* or earlier or prevent*) adj5 (assessment or detect* or diagnos* or discover* or evaluat* or exam* or onset or stage*)).ti,ab,kf.<br>26 or/21-25<br>27 (CRC adj3 (assessment* or screen* or detect* or diagnos* or discovery or exam* or endoscop*)).ti,ab,kf.<br>28 (15 or 20) and 26<br>29 27 or 28<br>30 ("Antigua and Barbuda" or "Antigua & Barbuda" or Argentin* or Aruba or Bahamas or Barbados or Belize or Belice or Bolivia* or Brazil* or Brasil* or Caribbean* or "Cayman Islands" or central-america* or Chile or chilean* or Colombia* or "Costa Rica*" or Cuba or cuban* or Curacao or Dominica* or "Dominican Republic" or Ecuador* or Salvador or Salvadorian* or French-Guiana or Grenada or Guadeloupe or Guatemala* or Guyana or Haiti* or Honduras or honduran* or Jamaica* or Latin-america* or latinoamerican* or latinamerican* or Martinique or Mexico or mexican* or Nicaragua* or Panama* or Paraguay* or Peru or Peruvian* or Puerto-ric* or boricua* or Salvadoran* or Sint-Maarten or south-america* or "Kitts & Nevis" or "Kitts and Nevis" or "Saint Lucia" or "St. Lucia" or "Saint Martin" or "St. Martin" or | Run on April 30, 2022, 664 citations retrieved. Rerun on February 27, 2023, 43 citations retrieved. |

© 2024 Montalvan-Sanchez EE et al. *JAMA Network Open.*

| | | |
|---|---|---|
| | "Vincent and the Grenadines" or "Vincent & the Grenadines" or Surinam* or "Trinidad and Tobago" or "Trinidad & Tobago" or "Turks and Caicos" or "Turks & Caicos" or Uruguay* or Venezuela* or "Virgin Islands" or "West Indies").tw,xm,kf.<br>31 29 and 30 | |
| Embase ( Elsevier) | #1 'colonoscopy'/exp<br>#2 'colonography, computed tomographic'/de<br>#3 'sigmoidoscopy'/de<br>#4 'occult blood'/de<br>#5 colonoscop*:ti,ab,kw<br>#6 colonograph*:ti,ab,kw<br>#7 sigmoidoscop*:ti,ab,kw<br>#8 ((colon OR colorectal OR 'colo rectal' OR colonic OR anal OR anus OR bowel OR rectocolonic* OR rectal OR rectum OR sigmoid*) NEAR/3 (endoscop* OR tomograph*)):ti,ab,kw<br>#9 ((occult OR hidden) NEAR/3 (blood OR bleeding OR haemorrhage* OR hemorrhage*)):ti,ab,kw<br>#10 ((stool OR fecal OR faecal OR faeces OR feces) NEAR/3 blood):ti,ab,kw<br>#11 hemoccult:ti,ab,kw<br>#12 fob:ti,ab,kw OR fobt*:ti,ab,kw OR gfobt*:ti,ab,kw OR ifobt*:ti,ab,kw<br>#13 ((stool OR fecal OR faecal OR faeces OR feces) NEAR/5 (immunologic* OR immunochem*)):ti,ab,kw<br>#14 ((stool OR guaiac OR guajac OR guaiacum) NEAR/5 (test OR sample*)):ti,ab,kw<br>#15 (((stool OR fecal OR faecal OR faeces OR feces) NEAR/3 (dna OR 'stool sdna')):ti,ab,kw) OR 'sdna fit':ti,ab,kw<br>#16 #1 OR #2 OR #3 OR #4 OR #5 OR #6 OR #7 OR #8 OR #9 OR #10 OR #11 OR #12 OR #13 OR #14 OR #15<br>#17 'colon cancer'/exp<br>#18 'colon tumor'/exp<br>#19 'colorectal tumor'/exp<br>#20 'colon polyp'/exp<br>#21 'colon disease'/de<br>#22 ((colon OR colorectal OR 'colo rectal' OR colonic OR anal OR anus OR bowel OR rectocolonic* OR rectal OR rectum OR sigmoid) NEAR/5 (cancer* OR carcinogen* OR neoplasm* OR neoplasia OR neoplastic OR adenoma* OR adenocarcinoma* OR polyp OR polyps OR carcinoma* OR malignan* OR tumor* OR tumour* OR precancer* OR premalignan*)):ti,ab,kw<br>#23 #17 OR #18 OR #19 OR #20 OR #21 OR #22<br>#24 'screening'/de<br>#25 'screening test'/de<br>#26 'mass screening'/de<br>#27 'early cancer diagnosis'/de<br>#28 'early diagnosis'/de<br>#29 'cancer diagnosis'/de<br>#30 screening*:ti,ab,kw OR screened:ti,ab,kw OR screens:ti,ab,kw<br>#31 ((early* OR earlier OR prevent*) NEAR/5 (assessment OR detect* OR diagnos* OR discover* OR evaluat* OR exam* OR onset OR stage*)):ti,ab,kw<br>#32 #24 OR #25 OR #26 OR #27 OR #28 OR #29 OR #30 OR 31<br>#33 ('crc' NEAR/3 (assessment* OR screen* OR detect* OR diagnos* OR discovery OR exam* OR endoscop*)):ti,ab,kw<br>#34 (#16 OR #23) AND #32<br>#35 #33 OR #34<br>#36 'south and central america'/exp | Run on April 30, 2022, 1992 citations retrieved. Rerun on February 27, 2023, 188 citations retrieved. |

© 2024 Montalvan-Sanchez EE et al. *JAMA Network Open.*

| | | |
|---|---|---|
| | #37 'caribbean islands'/exp<br>#38 'mexico'/exp<br>#39 'antigua and barbuda':ti,ab,kw OR 'antigua & barbuda':ti,ab,kw OR argentin*:ti,ab,kw OR aruba:ti,ab,kw OR bahamas:ti,ab,kw OR barbados:ti,ab,kw OR belize:ti,ab,kw OR belice:ti,ab,kw OR bolivia*:ti,ab,kw OR brazil*:ti,ab,kw OR brasil*:ti,ab,kw OR caribbean*:ti,ab,kw OR 'cayman islands':ti,ab,kw OR 'central america*':ti,ab,kw OR chile:ti,ab,kw OR chilean*:ti,ab,kw OR colombia*:ti,ab,kw OR 'costa rica*':ti,ab,kw OR cuba:ti,ab,kw OR cuban*:ti,ab,kw OR curacao:ti,ab,kw OR dominica*:ti,ab,kw OR 'dominican republic':ti,ab,kw OR ecuador*:ti,ab,kw OR salvador:ti,ab,kw OR salvadorian*:ti,ab,kw OR 'french guiana':ti,ab,kw OR grenada:ti,ab,kw OR guadeloupe:ti,ab,kw OR guatemala*:ti,ab,kw OR guyana:ti,ab,kw OR haiti*:ti,ab,kw OR honduras:ti,ab,kw OR honduran*:ti,ab,kw OR jamaica*:ti,ab,kw OR 'latin america*':ti,ab,kw OR latinoamerican*:ti,ab,kw OR latinamerican*:ti,ab,kw OR martinique:ti,ab,kw OR mexico:ti,ab,kw OR mexican*:ti,ab,kw OR nicaragua*:ti,ab,kw OR panama*:ti,ab,kw OR paraguay*:ti,ab,kw OR peru:ti,ab,kw OR peruvian*:ti,ab,kw OR 'puerto ric*':ti,ab,kw OR boricua*:ti,ab,kw OR salvadoran*:ti,ab,kw OR 'sint maarten':ti,ab,kw OR 'south america*':ti,ab,kw OR 'kitts & nevis':ti,ab,kw OR 'kitts and nevis':ti,ab,kw OR 'saint lucia':ti,ab,kw OR 'st. lucia':ti,ab,kw OR 'saint martin':ti,ab,kw OR 'st. martin':ti,ab,kw OR 'vincent and the grenadines':ti,ab,kw OR 'vincent & the grenadines':ti,ab,kw OR surinam*:ti,ab,kw OR 'trinidad and tobago':ti,ab,kw OR 'trinidad & tobago':ti,ab,kw OR 'turks and caicos':ti,ab,kw OR 'turks & caicos':ti,ab,kw OR uruguay*:ti,ab,kw OR venezuela*:ti,ab,kw OR 'virgin islands':ti,ab,kw OR 'west indies':ti,ab,kw<br>#40 #36 OR #37 OR #38 OR #39<br>#41 #35 AND #40 | |
| Google Scholar | allintitle: (CRC\|colorectal\|colon\|colorrectal\|colonoscopy\|colonoscopia) (screening\|tamizaje\|tamizar\|tamizacion\|rastreo\|rastreamento\|preventive\|prevention\|prevencion\|early\|temprano\|temprana) (America\|"Antigua and Barbuda"\|Argentina\|Aruba\|Brasil\|Brazil)<br><br>allintitle: (CRC\|colorectal\|colon\|colorrectal\|colonoscopy\|colonoscopia) (screening\|tamizaje\|tamizar\|tamizacion\|rastreo\|rastreamento\|preventive\|prevention\|prevencion\|early\|temprano\|temprana) (Bahamas\|Barbados\|Belize\|Belice\|Bolivia\|Caribbean\|"Cayman Islands")<br><br>allintitle: (CRC\|colorectal\|colon\|colorrectal\|colonoscopy\|colonoscopia) (screening\|tamizaje\|tamizar\|tamizacion\|rastreo\|rastreamento\|preventive\|prevention\|prevencion\|early\|temprano\|temprana) (Chile\|Colombia\|"Costa Rica"\|Cuba\|Curacao\|Dominica\|Dominican)<br><br>allintitle: (CRC\|colorectal\|colon\|colorrectal\|colonoscopy\|colonoscopia) (screening\|tamizaje\|tamizar\|tamizacion\|rastreo\|rastreamento\|preventive\|prevention\|prevencion\|early\|temprano\|temprana) (Ecuador\|Grenada\|Guadeloupe\|Guatemala\|Guyana\|Guiana)<br><br>allintitle: (CRC\|colorectal\|colon\|colorrectal\|colonoscopy\|colonoscopia) (screening\|tamizaje\|tamizar\|tamizacion\|rastreo\|rastreamento\|preventive\|prevention\|prevencion\|ea | Run on April 30, 2022, 119 citations retrieved after deduplication. Rerun on February 27, 2023, 16 citations retrieved. |

© 2024 Montalvan-Sanchez EE et al. *JAMA Network Open.*

| | | |
|---|---|---|
| | rly\|temprano\|temprana) (Haiti\|Honduras\|Jamaica\|Martinique\|Mexico\|Nicaragua\|Panama\|Paraguay\|Peru)<br><br>allintitle: (CRC\|colorectal\|colon\|colorrectal\|colonoscopy\|colonoscopia) (screening\|tamizaje\| tamizar\|tamizacion\|rastreo\|rastreamento\|preventive\|prevention\|prevencion\|early\|temprano\|temprana) ("Puerto Rico"\|Salvador\|Suriname\|Uruguay\|Sint-Maarten\|Kitts\|Nevis)<br><br>allintitle: (CRC\|colorectal\|colon\|colorrectal\|colonoscopy\|colonoscopia) (screening\|tamizaje\| tamizar\|tamizacion\|rastreo\|rastreamento\|preventive\|prevention\|prevencion\|early\|temprano\|temprana) (Lucia\|Saint Martin\|Grenadines\|Trinidad\|Tobago)<br><br>allintitle: (CRC\|colorectal\|colon\|colorrectal\|colonoscopy\|colonoscopia) (screening\|tamizaje\| tamizar\|tamizacion\|rastreo\|rastreamento\|preventive\|prevention\|prevencion\|early\|temprano\|temprana) (Turks\|Caicos\|Uruguay\|Venezuela\|"Virgin Islands"\|"West Indies") | |
| LILACS | LILACS https://lilacs.bvsalud.org/en/<br><br>("Cancer of Colon" OR "Cancer of the Colon" OR "Colon Cancer" OR "Colon Cancers" OR "Colon Neoplasm" OR "Colon Neoplasms" OR "Colonic Cancer" OR "Colonic Cancers" OR "Colonic Neoplasm" OR "Colonic Neoplasms" OR "Colorectal Cancer" OR "Colorectal Cancers" OR "cancer Colorrectal" OR "Colorectal adenocarcinoma" OR "adenocarcinoma colorectal" OR "adenocarcinoma colorretais" OR "colon adenocarcinoma" OR "adenocarcinoma de colon" OR "cancer Colorretal" OR "Colorectal Carcinoma" OR "Colorectal Carcinomas" OR "Carcinoma Colorrectal" OR "Carcinomas Colorectales" OR "Colorectal Neoplasm" OR "Colorectal Neoplasms" OR "Colorectal Neoplasias" OR "Neoplasms Colorectal" OR "Neoplasia Colorrectal" OR "Neoplasia Colorretal" OR "Neoplasias del Colon" OR "Neoplasias Colorretais" OR "Rectal neoplasm" OR "Rectal neoplasms" OR "Cancer of Rectum" OR "Cancer of the Rectum" OR "Rectal Cancer" OR "Rectal Cancers" OR "Rectal Tumor" OR "Rectal Tumors" OR "Rectum Cancer" OR "Rectum Cancers" OR "Rectum Neoplasm" OR "Rectum Neoplasms" OR " Neoplasias del Recto" OR "Neoplasias retais" OR "Colorectal Tumor" OR "Colorectal Tumors" OR "Anal Cancer" OR "Anal Cancers" OR "Anal Neoplasm" OR "Anal Neoplasms" OR "Anus Cancer" OR "Anus Cancers" OR "Anus Neoplasm" OR "Cancer of Anus" OR "Cancer of the Anus" OR "Neoplasias del Ano" OR "Neoplasias do Anus" OR "Neoplasias do Anus" OR "Tumor colorrectal" OR "Tumores colorrectales" OR "Neoplasias do Colo Sigmoide" OR "Cancer of Sigmoid" OR "Cancer of the Sigmoid" OR "Sigmoid Cancer" OR "Sigmoid Colon Cancer" OR "Sigmoid Neoplasm" OR "Sigmoidal Cancer" OR "Colonic Polyps" OR "Polipos del Colon" OR "Colonic Polyp" OR "Polipos do Colo" OR Colonoscopy OR Colonoscopia OR Colonography OR Colonografia OR Sigmoidoscopy OR "occult blood" OR "sangre oculta" OR "sangre en las heces" OR "sangre escondida" OR "sangue escondido" OR "Sangue Oculto" OR "sangue nas fezes" OR "Fecal Immunochemical" OR "inmunoquimica fecal" OR "stool DNA" OR sDNA OR "sDNA-FIT" OR hemoccult OR FOBT OR gFOBT OR iFOBT OR guaiac OR guajac OR guaiacum) AND (screening OR screenings OR tamizaje OR tamizar OR tamizacion OR rastreo OR rastreamento or rastreio OR "early detection" OR "deteccion temprana" OR "Deteccion Precoz" OR "Detecção Precoce" OR "early | Run on April 30, 2022, 1536 citations retrieved. Rerun on February 27, 2023, 40 citations retrieved. |

© 2024 Montalvan-Sanchez EE et al. *JAMA Network Open.*

| | | |
|---|---|---|
| | diagnosis" OR "diagnostico temprano" OR "Diagnostico Precoce" OR "early onset" OR "early assessment" OR "revision temprana" OR "chequeo temprano" OR "revision temprana" OR "revisao precoce" OR "early discovery" OR "descubrimiento temprano" OR "descoberta precoce" OR "avaliacao precoce" OR "preventive examination" OR "preventive evaluation" OR "evaluacion temprana" OR "evaluacion preventiva" OR "evaluacion temprana" OR "exames preventivos") | |
| PsycINFO 1887-current (EBSCO) | S1 colonoscop* OR colonograph* OR sigmoidoscop*<br>S2 ((colon OR colorectal OR colo-rectal OR colonic OR anal OR anus OR bowel OR rectocolonic* OR rectal OR rectum OR sigmoid* ) N3 (endoscop* OR tomograph* ))<br>S3 ((occult OR hidden) N3 (blood OR bleeding OR haemorrhage* OR hemorrhage* ))<br>S4 ((stool OR fecal OR faecal OR faeces OR feces ) N3 blood )<br>S5 hemoccult<br>S6 (FOB OR FOBT* OR gFOBT* OR iFOBT* )<br>S7 ((stool OR fecal OR faecal OR faeces OR feces ) N5 (immunologic* OR immunochem*))<br>S8 ((stool OR guaiac OR guajac OR guaiacum ) N5 (test OR sample*))<br>S9 (((stool OR fecal OR faecal OR faeces OR feces ) N3 (DNA OR sDNA )) OR sDNA-FIT)<br>S10 (S1 OR S2 OR S3 OR S4 OR S5 OR S6 OR S7 OR S8 OR S9)<br>S11 ((colon OR colorectal OR colo-rectal OR colonic OR anal OR anus OR bowel OR rectocolonic* OR rectal OR rectum OR sigmoid ) N5 (cancer* OR carcinogen* OR neoplasm* OR neoplasia OR neoplastic OR adenoma* OR adenocarcinoma* OR polyp OR polyps OR carcinoma* OR malignan* OR tumor* OR tumour* OR precancer* OR premalignan*))<br>S12 ( screening* OR screened OR screens)<br>S13 (( early* OR earlier OR prevent*) N5 (assessment OR detect* OR diagnos* OR discover* OR evaluat* OR exam* OR onset OR stage*))<br>S14 S12 OR S13<br>S15 ( CRC N3 (assessment* or screen* or detect* or diagnos* or discovery or exam* or endoscop*))<br>S16 (S10 OR S11) AND S14<br>S17 S15 OR S16<br>S18 TI ( "Antigua and Barbuda" or "Antigua & Barbuda" or Argentin* or Aruba or Bahamas or Barbados or Belize or Belice or Bolivia* or Brazil* or Brasil* or Caribbean* or "Cayman Islands" or central-america* or Chile or chilean* or Colombia* or "Costa Rica*" or Cuba or cuban* or Curacao or Dominica* or "Dominican Republic" or Ecuador* or Salvador or Salvadorian* or French-Guiana or Grenada or Guadeloupe or Guatemala* or Guyana or Haiti* or Honduras or honduran* or Jamaica* or Latin-america* or latinoamerican* or latinamerican* or Martinique or Mexico or mexican* or Nicaragua* or Panama* or Paraguay* or Peru or Peruvian* or Puerto-ric* or boricua* or Salvadoran* or Sint-Maarten or south-america* or "Kitts & Nevis" or "Kitts and Nevis" or "Saint Lucia" or "St. Lucia" or "Saint Martin" or "St. Martin" or "Vincent and the Grenadines" or "Vincent & the Grenadines" or Surinam* or "Trinidad and Tobago" or "Trinidad & Tobago" or "Turks and Caicos" or "Turks & Caicos" or Uruguay* or Venezuela* or "Virgin Islands" or "West Indies" ) OR SU ( "Antigua and Barbuda" or "Antigua & Barbuda" or Argentin* or Aruba or Bahamas or Barbados or Belize or Belice or Bolivia* or Brazil* or Brasil* or Caribbean* or "Cayman Islands" or central-america* or Chile or chilean* or Colombia* or "Costa Rica*" or Cuba or cuban* or Curacao or Dominica* or "Dominican Republic" or Ecuador* or Salvador or Salvadorian* or French-Guiana or Grenada or Guadeloupe or Guatemala* or | Run on April 30, 2022, 57 citations retrieved. Rerun on February 27, 2023, 1 citation retrieved |

| | | |
|---|---|---|
| | Guyana or Haiti* or Honduras or honduran* or Jamaica* or Latin-america* or latinoamerican* or latinamerican* or Martinique or Mexico or mexican* or Nicaragua* or Panama* or Paraguay* or Peru or Peruvian* or Puerto-ric* or boricua* or Salvadoran* or Sint-Maarten or south-america* or "Kitts & Nevis" or "Kitts and Nevis" or "Saint Lucia" or "St. Lucia" or "Saint Martin" or "St. Martin" or "Vincent and the Grenadines" or "Vincent & the Grenadines" or Surinam* or "Trinidad and Tobago" or "Trinidad & Tobago" or "Turks and Caicos" or "Turks & Caicos" or Uruguay* or Venezuela* or "Virgin Islands" or "West Indies" ) OR PL ( "Antigua and Barbuda" or "Antigua & Barbuda" or Argentin* or Aruba or Bahamas or Barbados or Belize or Belice or Bolivia* or Brazil* or Brasil* or Caribbean* or "Cayman Islands" or central-america* or Chile or chilean* or Colombia* or "Costa Rica*" or Cuba or cuban* or Curacao or Dominica* or "Dominican Republic" or Ecuador* or Salvador or Salvadorian* or French-Guiana or Grenada or Guadeloupe or Guatemala* or Guyana or Haiti* or Honduras or honduran* or Jamaica* or Latin-america* or latinoamerican* or latinamerican* or Martinique or Mexico or mexican* or Nicaragua* or Panama* or Paraguay* or Peru or Peruvian* or Puerto-ric* or boricua* or Salvadoran* or Sint-Maarten or south-america* or "Kitts & Nevis" or "Kitts and Nevis" or "Saint Lucia" or "St. Lucia" or "Saint Martin" or "St. Martin" or "Vincent and the Grenadines" or "Vincent & the Grenadines" or Surinam* or "Trinidad and Tobago" or "Trinidad & Tobago" or "Turks and Caicos" or "Turks & Caicos" or Uruguay* or Venezuela* or "Virgin Islands" or "West Indies" ) OR AB ( "Antigua and Barbuda" or "Antigua & Barbuda" or Argentin* or Aruba or Bahamas or Barbados or Belize or Belice or Bolivia* or Brazil* or Brasil* or Caribbean* or "Cayman Islands" or central-america* or Chile or chilean* or Colombia* or "Costa Rica*" or Cuba or cuban* or Curacao or Dominica* or "Dominican Republic" or Ecuador* or Salvador or Salvadorian* or French-Guiana or Grenada or Guadeloupe or Guatemala* or Guyana or Haiti* or Honduras or honduran* or Jamaica* or Latin-america* or latinoamerican* or latinamerican* or Martinique or Mexico or mexican* or Nicaragua* or Panama* or Paraguay* or Peru or Peruvian* or Puerto-ric* or boricua* or Salvadoran* or Sint-Maarten or south-america* or "Kitts & Nevis" or "Kitts and Nevis" or "Saint Lucia" or "St. Lucia" or "Saint Martin" or "St. Martin" or "Vincent and the Grenadines" or "Vincent & the Grenadines" or Surinam* or "Trinidad and Tobago" or "Trinidad & Tobago" or "Turks and Caicos" or "Turks & Caicos" or Uruguay* or Venezuela* or "Virgin Islands" or "West Indies" ) OR KW ( "Antigua and Barbuda" or "Antigua & Barbuda" or Argentin* or Aruba or Bahamas or Barbados or Belize or Belice or Bolivia* or Brazil* or Brasil* or Caribbean* or "Cayman Islands" or central-america* or Chile or chilean* or Colombia* or "Costa Rica*" or Cuba or cuban* or Curacao or Dominica* or "Dominican Republic" or Ecuador* or Salvador or Salvadorian* or French-Guiana or Grenada or Guadeloupe or Guatemala* or Guyana or Haiti* or Honduras or honduran* or Jamaica* or Latin-america* or latinoamerican* or latinamerican* or Martinique or Mexico or mexican* or Nicaragua* or Panama* or Paraguay* or Peru or Peruvian* or Puerto-ric* or boricua* or Salvadoran* or Sint-Maarten or south-america* or "Kitts & Nevis" or "Kitts and Nevis" or "Saint Lucia" or "St. Lucia" or "Saint Martin" or "St. Martin" or "Vincent and the Grenadines" or "Vincent & the Grenadines" or Surinam* or "Trinidad and Tobago" or "Trinidad & Tobago" or "Turks and Caicos" or "Turks & Caicos" or Uruguay* or Venezuela* or "Virgin Islands" or "West Indies" )<br>S19 (S17 AND S18) | |
| SciELO | ("Cancer of Colon" OR "Cancer of the Colon" OR "Colon Cancer" OR "Colon Cancers" OR "Colon Neoplasm" OR "Colon Neoplasms" OR "Colonic Cancer" OR "Colonic Cancers" OR "Colonic Neoplasm" OR "Colonic Neoplasms" OR "Colorectal Cancer" OR "Colorectal Cancers" OR "cancer | Run on April 29, 2022, 362 citations |

© 2024 Montalvan-Sanchez EE et al. *JAMA Network Open.*

| | | |
|---|---|---|
| | Colorrectal" OR "Colorectal adenocarcinoma" OR "adenocarcinoma colorectal" OR "adenocarcinoma colorretais" OR "colon adenocarcinoma" OR "adenocarcinoma de colon" OR "cancer Colorretal"  OR "Colorectal Carcinoma" OR "Colorectal Carcinomas" OR "Carcinoma Colorrectal" OR "Carcinomas Colorectales" OR "Colorectal Neoplasm" OR "Colorectal Neoplasms" OR "Colorectal Neoplasias" OR "Neoplasms Colorectal" OR "Neoplasia Colorrectal" OR "Neoplasia Colorretal" OR "Neoplasias del Colon" OR "Neoplasias Colorretais" OR "Rectal neoplasm" OR "Rectal neoplasms" OR "Cancer of Rectum" OR  "Cancer of the Rectum" OR "Rectal Cancer" OR "Rectal Cancers" OR "Rectal Tumor" OR "Rectal Tumors" OR "Rectum Cancer" OR "Rectum Cancers" OR "Rectum Neoplasm" OR "Rectum Neoplasms" OR " Neoplasias del Recto" OR "Neoplasias retais" OR "Colorectal Tumor" OR "Colorectal Tumors" OR "Anal Cancer" OR "Anal Cancers" OR "Anal Neoplasm" OR "Anal Neoplasms" OR "Anus Cancer" OR "Anus Cancers" OR "Anus Neoplasm" OR "Cancer of Anus" OR "Cancer of the Anus" OR "Neoplasias del Ano" OR "Neoplasias do Anus" OR "Neoplasias do Anus" OR "Tumor colorrectal" OR "Tumores colorrectales" OR "Neoplasias do Colo Sigmoide" OR "Cancer of Sigmoid" OR "Cancer of the Sigmoid" OR "Sigmoid Cancer" OR "Sigmoid Colon Cancer" OR "Sigmoid Neoplasm" OR "Sigmoidal Cancer" OR "Colonic Polyps" OR "Polipos del Colon" OR "Colonic Polyp" OR "Polipos do Colo" OR Colonoscopy OR Colonoscopia OR Colonography OR Colonografia OR Sigmoidoscopy OR "occult blood" OR "sangre oculta" OR "sangre en las heces" OR "sangre escondida" OR "sangue escondido" OR "Sangue Oculto" OR "sangue nas fezes" OR "Fecal Immunochemical" OR "inmunoquimica fecal" OR "stool DNA" OR sDNA OR "sDNA-FIT" OR hemoccult OR FOBT OR gFOBT OR iFOBT OR guaiac OR guajac OR guaiacum)  AND (screening OR screenings OR tamizaje OR tamizar OR tamizacion OR rastreo OR rastreamento or rastreio OR "early detection" OR "deteccion temprana" OR "Deteccion Precoz" OR "Detecção Precoce" OR "early diagnosis" OR "diagnostico temprano" OR "Diagnostico Precoce" OR "early onset" OR "early assessment" OR "revision temprana" OR "chequeo temprano" OR "revision temprana" OR "revisao precoce" OR "early discovery" OR "descubrimiento temprano" OR "descoberta precoce" OR "avaliacao precoce" OR "preventive examination" OR "preventive evaluation" OR "evaluacion temprana" OR "evaluacion preventiva" OR "evaluacion temprana" OR "exames preventivos") | retrieved. Rerun on February 27, 2023, 25 citations retrieved |
| Web of Science Core Collection | ((((TS=(colonoscop* OR colonograph* OR sigmoidoscop* ) OR TS=((colon OR colorectal OR colo-rectal OR colonic OR anal OR anus OR bowel OR rectocolonic* OR rectal OR rectum OR sigmoid* ) NEAR/3 (endoscop* OR tomograph* )) OR TS=((occult OR hidden) NEAR/3 (blood OR bleeding OR haemorrhage* OR hemorrhage* )) OR TS=((stool OR fecal OR faecal OR faeces OR feces ) NEAR/3 blood ) OR TS=hemoccult OR TS=(FOB OR FOBT* OR gFOBT* OR iFOBT* )) OR TS=((colon OR colorectal OR colo-rectal OR colonic OR anal OR anus OR bowel OR rectocolonic* OR rectal OR rectum OR sigmoid ) NEAR/5 (cancer* or carcinogen* or neoplasm* or neoplasia or neoplastic or adenoma* or adenocarcinoma* or polyp or polyps or carcinoma* or malignan* or tumor* or tumour* or precancer* or premalignan*)))) AND (TS=( screening* OR screened OR screens) OR TS=(( early* OR earlier OR prevent*) NEAR/5 (assessment or detect* or diagnos* or discover* OR evaluat* OR exam* or onset or stage*assessment or detect* or diagnos* or discover* or exam* or onset or stage*))) OR TS=( CRC NEAR/3 (assessment* or screen* or detect* or diagnos* or discovery or exam* or endoscop*))) AND  TS= ("Antigua and Barbuda" or "Antigua & Barbuda" or Argentin* or Aruba or Bahamas or Barbados or Belize or Belice or Bolivia* or Brazil* or Brasil* or Caribbean* or "Cayman Islands" or central- | Run on April 28, 2022, 581 citations retrieved. Rerun on February 27, 2023, 37 citations retrieved |

© 2024 Montalvan-Sanchez EE et al. *JAMA Network Open.*

| | | |
|---|---|---|
| | america* or Chile or chilean* or Colombia* or "Costa Rica*" or Cuba or cuban* or Curacao or Dominica* or "Dominican Republic" or Ecuador* or Salvador or Salvadorian* or French-Guiana or Grenada or Guadeloupe or Guatemala* or Guyana or Haiti* or Honduras or honduran* or Jamaica* or Latin-america* or latinoamerican* or latinamerican* or Martinique or Mexico or mexican* or Nicaragua* or Panama* or Paraguay* or Peru or Peruvian* or Puerto-ric* or boricua* or Salvadoran* or Sint-Maarten or south-america* or "Kitts & Nevis" or "Kitts and Nevis" or "Saint Lucia" or "St. Lucia" or "Saint Martin" or "St. Martin" or "Vincent and the Grenadines" or "Vincent & the Grenadines" or Surinam* or "Trinidad and Tobago" or "Trinidad & Tobago" or "Turks and Caicos" or "Turks & Caicos" or Uruguay* or Venezuela* or "Virgin Islands" or "West Indies") | |

© 2024 Montalvan-Sanchez EE et al. *JAMA Network Open.*

**eFigure 1. Flow Diagram of Study Inclusion**

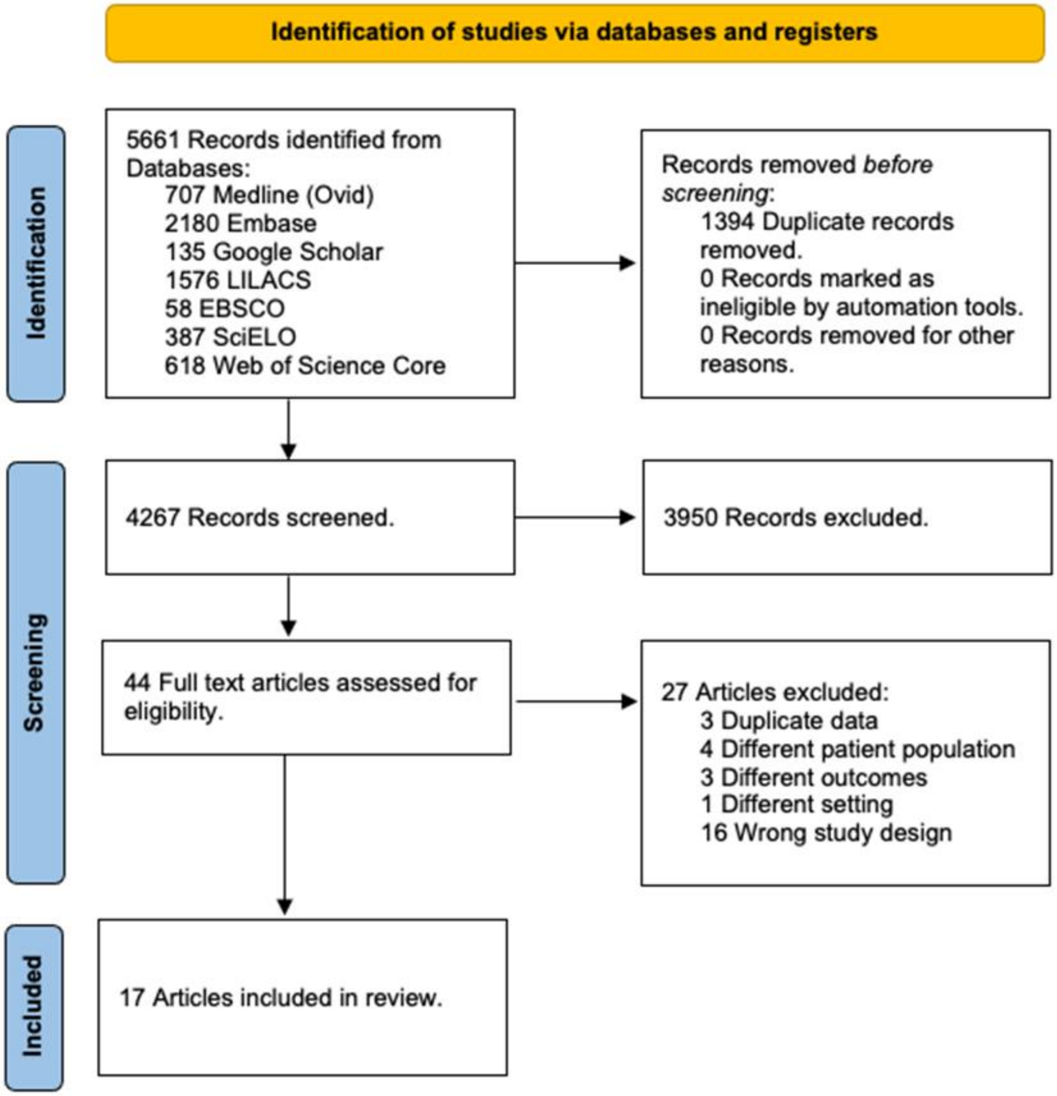


© 2024 Montalvan-Sanchez EE et al. *JAMA Network Open.*

**eTable 2. Excluded studies and reasons for exclusion.**

| |
|---|
| Bandres D, Yanes E, Caravajal A, et al. Results of a large screening program for colonic polyps and colon cancer using flexible sigmoidoscopy in Venezuela. *Gastroenterology*. 1997;112(4):A535-A535. |
| Reason for exclusion: Wrong study design |
| |
| Bravo Hernández N, Gómez Lloga T, Noa Garbey M, Quevedo Navarro AL, Gómez Lloga R. Pesquisa de cáncer de colon en grupos de riesgos del Policlínico Universitario “Omar Ranedo Pubillones de Guantánamo. *Revista Información Científica*. 2018;97(1):95-105. |
| Reason for exclusion: Wrong study design |
| |
| Carballo EN, Melendez FDH. Prevalence of advanced colorectal neoplasia according to risk categories at screening colonoscopy in a tertiary referral center. *Journal of Clinical Oncology*. 2018;36(4)doi:10.1200/JCO.2018.36.4-suppl.578 |
| Reason for exclusion: Wrong study design |
| |
| Casal ER, Velázquez EN, Mejía RM, Cuneo A, Pérez-Stable EJ. Rastreo del cáncer colorrectal: Conocimiento y actitud de la población. *Medicina (BAires)*. 2009;69(1):135-142. |
| Reason for exclusion: Wrong study design |
| |
| Cawich SO, Phillips E, Moore S, Ramkissoon S, Padmore G, Griffith S. Colorectal cancer in an Eastern Caribbean nation: are we missing an opportunity for secondary prevention? *Revista Panamericana de Salud Pública*. 2022;46 |
| Reason for exclusion: Wrong study design |
| |
| Chandra A, Cooper E. Colorectal cancer in the bahamian population. *American Journal of Gastroenterology*. 2014;109:S618. doi:10.1038/ajg.2014.285 |
| Reason for exclusion: Different outcome |
| |
| Coughlin SS, Lubetkin EI, Hay JL, Raphael R, Smith SA. Promoting colorectal cancer screening among Haitian Americans. *Journal of the Georgia Public Health Association*. 2015;5(2):149-152. |
| Reason for exclusion: Wrong study design |
| |
| Estefanía D, Tyrrell C, Bugallo F, et al. Pesquisa del cáncer colorrectal en un hospital de comunidad: experiencia, resultados y eficacia del método. *Rev argent coloproctología*. 2011;22(1):10-15. |
| Reason for exclusion: Wrong study design |
| |

© 2024 Montalvan-Sanchez EE et al. *JAMA Network Open.*

| |
|---|
| Fernandez JL, Gallegos M, Brochero A, Arevalo C, Piccioni H, Gutierrez Galiana H. [Screening for colorectal cancer with an immunological fecal occult blood test]. *Pesquisa del cancer colorrectal con una prueba inmunologica para sangre oculta en material fecal*. 1999;29(2):73-8. |
| Reason for exclusion: Wrong study design |
| |
| Garza AA, Becerra CA, Gonzalez-Gonzalez JA, et al. Colorectal cancer screening in Mexico: The first one hundred patients. *American Journal of Gastroenterology*. 2006;101(9):S559-S559. doi:10.14309/00000434-200609001-01471 |
| Reason for exclusion: Wrong study design |
| |
| Jimenez G, Perez MdlA, Vega H, Hano O. Pesquisaje de lesiones precancerosas y cancerosas del colon en los trabajadores del asbesto. *Rev cuba hig epidemiol*. 1995;33(1/2):25-30. |
| Reason for exclusion: Different patient population |
| |
| Kennedy LS, Marra KA, Marshall LaRochelle EP, et al. Cancer screening in rural Honduras: Maximizing impact with a multiorgan screening approach. *Cancer Epidemiology Biomarkers and Prevention*. 2018;27(7)doi:10.1158/1538-7755.DISP17-B20 |
| Reason for exclusion: Wrong study design |
| |
| Lasser KE, Murillo J, Lisboa S, et al. Colorectal cancer screening among ethnically diverse, low-income patients: a randomized controlled trial. *Archives of Internal Medicine*. 2011;171(10):906-12. doi:10.1001/archinternmed.2011.201 |
| Reason for exclusion: Wrong study design |
| |
| Lathroum L, Ramos-Mercado F, Villafana M, Cruz-Correa MR. Prevalence of colorectal neoplasia in asymptomatic hispanics undergoing screening colonoscopy. *Gastrointestinal Endoscopy*. 2010;71(5):AB194. doi:10.1016/j.gie.2010.03.333 |
| Reason for exclusion: Wrong study design |
| |
| Lopez-Kostner F, Fullerton DA, Kronberg U, et al. Screening colonoscopy among first degree relatives of patients with colorectal carcinoma. *Revista Medica De Chile*. 2006;134(8):997-1001. |
| Reason for exclusion: Different patient population |
| |
| Montano D, Fenocchi Môneda ER, Martínez Gómez L, Tolve Tilbe JA, Rondán Olivera M. Programa de Screening en cáncer colo-rectal. *Cir Urug*. 2005;75(3):180-188. |
| Reason for exclusion: Wrong study design |
| |
| Montano Morgade DE, Fenocchi Moneda ER, Martínez Gómez L, Rondán Olivera M, Olano Gossweiler AC. Screening en cáncer colorrectal. Primer estudio de población asintomática en Uruguay. *Cir Urug*. 2000:14-9. |
| Reason for exclusion: Duplicate data |

© 2024 Montalvan-Sanchez EE et al. *JAMA Network Open.*

| |
|---|
| |
| Nery TDD, Hara EKM, Feijo CD, et al. Occupational Health As a Tool to Improve Workers' Health. Experience Report in Colorectal Cancer Screening in the Approach of 12,233 Professionals in the Largest Hospital Complex in Latin America. *Safety and Health at Work*. 2022;13:S235-S235. |
| Reason for exclusion: Wrong study design |
| |
| Pagan-Torres H, Borrero WM, Soto M, Cruz-Correa MR. EVALUATION OF THE DISSEMINATION AND IMPLEMENTATION OF A NATIONAL COLORECTAL CANCER SCREENING PROGRAM AMONG PRIMARY CARE PHYSICIANSIN PUERTO RICO. *Gastroenterology*. 2019;156(6):S809-S809. |
| Reason for exclusion: Wrong study design |
| |
| Pozo Parilli JC, Méndez Castro M, Pinto Plata V, Gómez LG, Ott Itriago S. Tumores de colon: experiecnia en un Centro Clínico de Caracas. *Gen*. 1994;48(1):19-24. |
| Reason for exclusion: Different setting |
| |
| Rendon RF, Carrillo YB, Jacques J, et al. SCREENING COLONOSCOPY IN A NORTHWESTERN POPULATION IN MEXICO: BARRIERS AND FACTORS RELATED TO THE PROCEDURE IN AVERAGE RISK PATIENTS. *Gastroenterology*. 2017;152(5):S529-S529. |
| Reason for exclusion: Different outcome |
| |
| Rivera G, Imbert AL, Bird J, et al. Yield of Fecal Immunohistochemical Test (FIT) for Colorectal Cancer Screening at the VA Caribbean Healthcare System: 1949. *Official journal of the American College of Gastroenterology\| ACG*. 2012;107:S794. |
| Reason for exclusion: Different patient population |
| |
| Rivera G, Imbert AL, Bird J, et al. Yield of Fecal Immunohistochemical Test (FIT) for Colorectal Cancer Screening at the VA Caribbean Healthcare System. *American Journal of Gastroenterology*. 2012;107:S794-S794. doi:10.14309/00000434-201210001-01949 |
| Reason for exclusion: Duplicate data |
| |
| Silva ACB, Vicentini MFB, Mendoza EZ, et al. Young-age onset colorectal cancer in Brazil: Analysis of incidence, clinical features, and outcomes in a tertiary cancer center. *Current Problems in Cancer*. 2019;43(5):477-486. doi:10.1016/j.currproblcancer.2019.01.009 |
| Reason for exclusion: Different outcome |
| |
| Tchekmedyian A, Messuti A, Richelli R, et al. Colorectal Cancer Screening and Post-polypectomy Follow-up. Results from a National Survey in Uruguay: 1467. *Official journal of the American College of Gastroenterology\| ACG*. 2009;104:S551-S552. |
| Reason for exclusion: Different patient population |
| |

© 2024 Montalvan-Sanchez EE et al. *JAMA Network Open.*

| |
|---|
| Tchekmedyian A, Messuti A, Richelli R, et al. Colorectal Cancer Screening and Post-polypectomy Follow-up. Results from a National Survey in Uruguay. *American Journal of Gastroenterology*. 2009;104:S551-S552. doi:10.14309/00000434-200910003-01467 |
| Reason for exclusion: Duplicate data |
| |
| Vega Sánchez H, Jiménez Mesa G, González-Carbajal M, Grá Ormas B. Pesquisaje de lesiones premalignas y malignas de colon mediante sangre oculta en heces fecales. *Rev cuba cir*. 1993;32(2):131-7. |
| Reason for exclusion: Wrong study design |
| |

Wrong study design: Studies that do not meet the specific design requirements outlined in the inclusion criteria (i.e. Studies focusing in general colorectal health without specifically focusing on screening programs, or incidence, mortality and survival studies).

© 2024 Montalvan-Sanchez EE et al. *JAMA Network Open.*

## eTable 3. Risk of bias using the New Castle Ottawa Scale for Comparative Studies.

| First Author | Year | Country | Adapted NOS quality assessment. | | | Total Score | Risk of bias |
|---|---|---|---|---|---|---|---|
| | | | Selection | Comparability | Exposure or outcome | | |
| Fenocchi et al. | 2006 | Uruguay | **** | ** | *** | 9 | Low |
| Garcia-Osogobio et al. | 2015 | Mexico | **** | * | *** | 8 | Low |
| Galvez-Rios et al. | 2020 | Mexico | **** | * | ** | 7 | Medium |
| Averbach et al. | 2021 | Brazil | **** | ** | ** | 8 | Low |
| Braga et al. | 2017 | Brazil | ** | * | *** | 6 | Medium |
| Ruiz et al. | 2021 | Argentina | **** | * | *** | 8 | Low |
| Silva et al. | 2011 | Chile | ** | * | *** | 6 | Medium |
| Teixeira et al. | 2017 | Brazil | *** | * | *** | 7 | Medium |
| Okada et al. | 2015 | Chile | **** | * | *** | 8 | Low |
| Guimaraes et al. | 2021 | Brazil | **** | ** | *** | 9 | Low |
| Lopez-Kostner et al. | 2012 | Chile | **** | * | *** | 8 | Low |
| Alfaro-Seguro et al. | 2020 | Costa Rica | **** | ** | ** | 9 | Low |
| Fernandez et al. | 2021 | Argentina | **** | * | ** | 7 | Medium |
| Manzano-Robleda et al. | 2020 | Mexico | **** | ** | ** | 8 | Low |
| Remes-Troche et al. | 2020 | Mexico | **** | * | ** | 7 | Medium |
| Rettally | 2008 | Panama | **** | * | ** | 7 | Medium |
| Fenocchi et al. | 2015 | Uruguay | **** | ** | ** | 8 | Low |

Note: The components listed above generate a total sum score for assessing risk of bias for each study. Total sum scores range from 0 to 9 points. Studies were grouped into 'low risk of bias' (8-9 points), 'moderate risk of bias' (5-7 points) or 'high risk of bias' (1-4 points). Comparability specifically referred to comparability between subjects in both outcome groups—i.e. control for confounding. We retained the original language from validation studies, even though all studies in our review only had one intervention group (without comparison group). The selection category also did not include the 'ascertainment of exposure' portion, because all studies included only intervention arms (no control groups).

© 2024 Montalvan-Sanchez EE et al. *JAMA Network Open.*

## eTable 4. Strategies for sampling and recruitment of included studies.

*Recruitment modes: Personal – direct interaction between study or healthcare personnel and potential subjects “face-to-face interaction”; Non-personal – not requiring direct face-to-face interaction [§]

| **Study characteristics** | | | | **Sampling** | | | **Recruitment** | | | | |
|---|---|---|---|---|---|---|---|---|---|---|---|
| **Author** | **Year** | **Country** | **Study type** | **Sampling** | **Eligibility age** | **Risk of CRC** | **Recruitment location** | **Recruitment Mode[b]** | **Face-face** | **Letter/ e-mail** | **Media** |
| **Fenocchi et al.** | 2006 | Uruguay | Prospective | Voluntary response | >50 | Average | Community | Media | | | X |
| **Rettally** | 2008 | Panama | Retrospective | Convenience | >50 | Increased | Community | NR | | | |
| **Silva et al.** | 2011 | Chile | Prospective | Voluntary response | >50 | Increased | Community | Personal/Media | | X | X |
| **Lopez-Kostner et al.** | 2012 | Chile | Prospective | Voluntary response | >50 | Average | CHC (Clinic) | Personal | X | | |
| **Fenocchi et al.** | 2015 | Uruguay | Prospective | Population-based | >50 | Average[a] | Hospital | NR | | | |
| **Garcia-Osogobio et al.** | 2015 | Mexico | Prospective | Convenience | 40-79 | Average | Workplace | Media | | | X |
| **Braga et al.** | 2017 | Brazil | Cross-sectional | Population-based | 50-70 | Elevated | CHC (Clinic) | Personal | X | | |
| **Teixeira et al.** | 2017 | Brazil | Prospective | Population-based | 50-75 | Average[a] | CHC (Clinic) | Personal | X | | |
| **Alfaro-Seguro et al.** | 2020 | Costa Rica | Prospective | Population-based | 50-75 | Average | Community | Personal | X | | |
| **Galvez-Rios et al.** | 2020 | Mexico | Prospective | Voluntary response | >50 | Average[a] | Community | Media | | | X |
| **Manzano-Robleda et al.** | 2020 | Mexico | Prospective | Convenience | 50-75 | Average | Workplace | Media | | | X |
| **Okada et. Al** | 2020 | Chile | Prospective | Population-based | NR | Increased | Hospital | Personal/Media | | | X |
| **Remes-Troche et al.** | 2020 | Mexico | Prospective | Voluntary response | >50 | Average | Community | Media | | | X |
| **Averbach et al.** | 2021 | Brazil | Prospective | Population-based | 50-70 | Average | Hospital | Personal | X | | |
| **Fernandez et al.** | 2021 | Argentina | Prospective | Convenience | 50-75 | Average | Hospital | Personal/Media | X | | X |
| **Guimaraes et al.** | 2021 | Brazil | Cross-sectional | Convenience | 50-65 | Average | CHC and Health Fairs | Personal | X | | |
| **Ruiz et al.** | 2021 | Argentina | Retrospective | Convenience | 50-75 | Average[a] | CHC (Clinic) | Personal | X | | |

Family history Not reported or effort not made to exclude. [b] Recruitment strategies included media advertisement only (n=5), personal invitation (n=7), combination of personal and media (n=3), and unspecified (n=2). Media modes of recruitment included radio and TV advertisements, posters in public and private institutions, and brochure distribution. “Personal” recruitment was carried out by healthcare professionals, community health workers/ navigators (CHW), or researchers, via in-person encounters or using individually addressed letters and/or emails. In-person encounters included clinics or hospitals (opportunistically when they visited for reasons other than screening; n= 9), or community settings (public places and own homes [n=6] or workplace [n=2]).

© 2024 Montalvan-Sanchez EE et al. *JAMA Network Open.*

**eTable 5. Univariate Meta regression**

| | Adenoma Detection Rate | | | Advanced Adenoma Detection Rate | | | CRC Detection Rate | | |
|---|---|---|---|---|---|---|---|---|---|
| **Moderator** | Coefficient | 95% CI | p-value | Coefficient | 95% CI | p-value | Coefficient | 95% CI | p-value |
| Age (mean) | 0.004 | -0.071 to 0.078 | 0.926 | 0.090 | -0.043 to 0.224 | 0.185 | **0.045** | **0.015 to 0.076** | **0.004** |
| CRC Family History (%) | -0.002 | -0.010 to 0.007 | 0.670 | -0.026 | -0.0719 to 0.191 | 0.256 | -0.0011 | -0.005 to 0.005 | 0.963 |
| Male (%) | **-0.032** | **-0.057 to -0.007** | **0.011** | **-0.036** | **-0.052 to -0.212** | **<0.001** | -0.010 | -0.026 to 0.007 | 0.243 |
| Sample Size | | | | | | | | | |
| Sample Size 1000-10,000 | **0.483** | **0.027 to 0.937** | **0.038** | 0.020 | -0.556 to 0.597 | 0.945 | -0.004 | -0.039 to 0.382 | 0.984 |
| Sample Size >10,000 | -0.146 | -0.594 to 0.302 | 0.523 | -0.183 | -0.753 to 0.388 | 0.533 | -0.055 | -0.433 to 0.325 | 0.775 |
| Participation Rate | 0.013 | -0.002 to 0.029 | 0.086 | 0.007 | -0.009 to 0.022 | 0.390 | 0.002 | -0.008 to 0.013 | 0.681 |
| FIT Positivity Rate | -0.023 | -0.058 to 0.123 | 0.202 | -0.003 | -0.042 to 0.035 | 0.877 | 0.005 | -0.019 to 0.029 | 0.771 |
| Colonoscopy follow-up | 0.006 | -0.003 to 0.016 | 0.206 | -0.001 | -0.003 to 0.001 | 0.193 | -0.001 | -0.002 to 0.001 | 0.073 |
| Publication year | -0.009 | -0.056 to 0.036 | 0.677 | -0.006 | -0.041 to 0.294 | 0.732 | 0.001 | -0.022 to 0.025 | 0.928 |
| Region (South America) | 0.118 | -0.330 to 0.567 | 0.605 | -0.104 | -0.499 to 0.291 | 0.606 | -0.119 | -0.359 to 0.120 | 0.328 |
| FIT cut-off | | | | | | | | | |
| 50ng/ml | 0.337 | -0.267 to 0.941 | 0.274 | **0.596** | **0.036 to 1.155** | **0.037** | 0.182 | -0.168 to 0.533 | 0.307 |
| 100ng/ml | 0.237 | -0.367 to 0.842 | 0.442 | 0.423 | -0.145 to 0.992 | 0.144 | 0.057 | -0.293 to 0.409 | 0.747 |
| Funding | | | | | | | | | |
| Governmental | Ref | | | Ref | | | Ref | | |
| Private | 0.447 | -0.237 to 1.132 | 0.200 | 0.453 | -0.288 to 1.193 | 0.231 | -0.056 | -0.412 to 0.298 | 0.754 |
| International | -0.036 | -0.561 to 0.489 | 0.892 | 0.251 | -0.287 to 0.788 | 0.361 | 0.126 | -0.312 to 0.565 | 0.574 |
| Risk of Bias: Medium | -0.195 | -0.619 to 0.229 | 0.368 | -0.037 | -0.444 to 0.369 | 0.857 | 0.035 | -0.206 to 0.277 | 0.772 |

© 2024 Montalvan-Sanchez EE et al. *JAMA Network Open.*

## eFigure 2. Participation Rate (FIT Given/FIT Returned) by region.

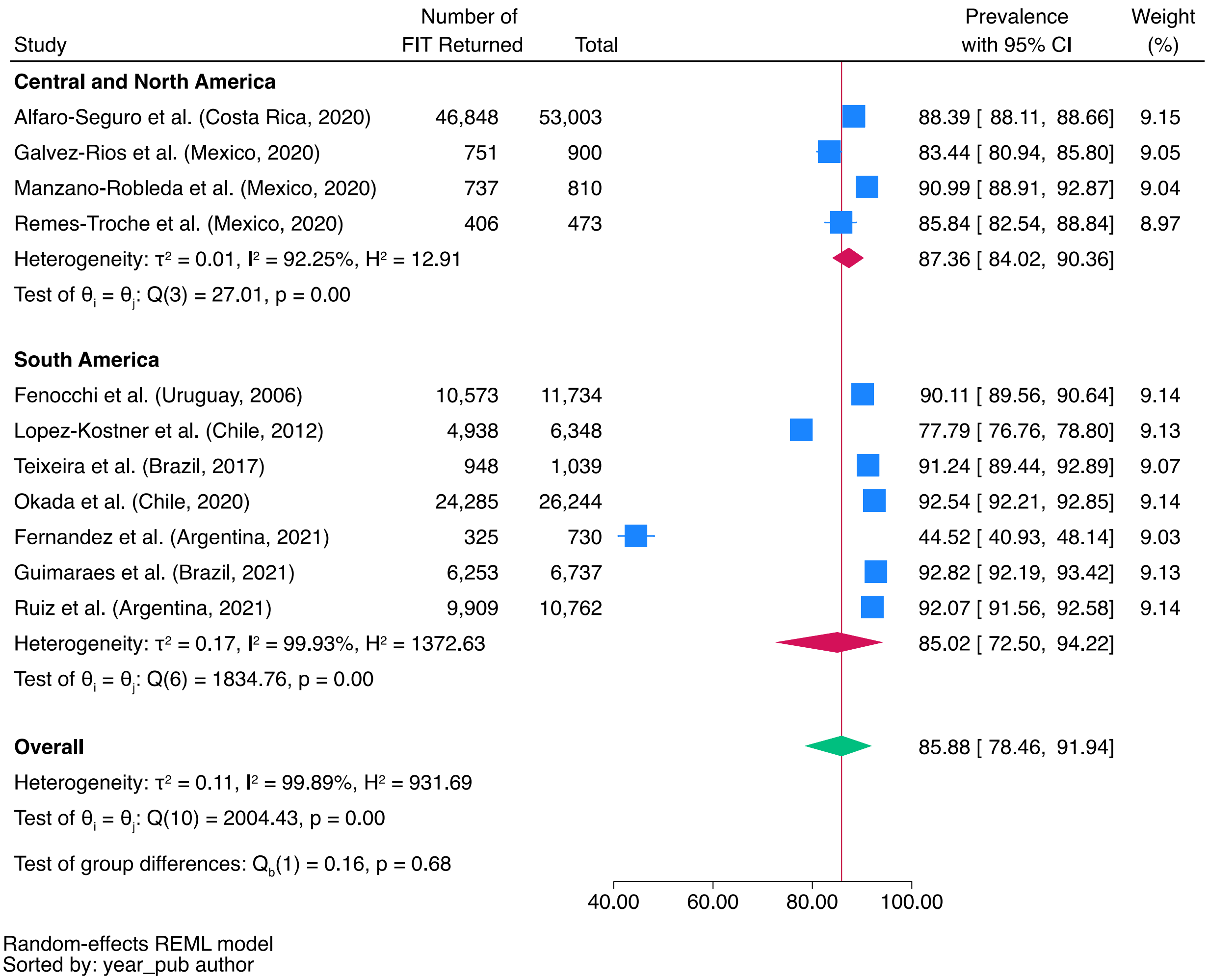


© 2024 Montalvan-Sanchez EE et al. *JAMA Network Open.*

## eFigure 3. Participation Rate (FIT Given/FIT Returned) by Recruitment strategy

| Study | Number of FIT Returned | Total | Prevalence with 95% CI | Weight (%) |
|---|---|---|---|---|
| **Media** | | | | |
| Fenocchi et al. (Uruguay, 2006) | 10,573 | 11,734 | 90.11 [ 89.56, 90.64] | 9.14 |
| Galvez-Rios et al. (Mexico, 2020) | 751 | 900 | 83.44 [ 80.94, 85.80] | 9.05 |
| Manzano-Robleda et al. (Mexico, 2020) | 737 | 810 | 90.99 [ 88.91, 92.87] | 9.04 |
| Remes-Troche et al. (Mexico, 2020) | 406 | 473 | 85.84 [ 82.54, 88.84] | 8.97 |
| Heterogeneity: $\tau^2 = 0.01$, $I^2 = 93.31\%$, $H^2 = 14.95$ | | | 87.85 [ 84.15, 91.11] | |
| Test of $\theta_i = \theta_j$: Q(3) = 41.34, p = 0.00 | | | | |
| **Personal** | | | | |
| Lopez-Kostner et al. (Chile, 2012) | 4,938 | 6,348 | 77.79 [ 76.76, 78.80] | 9.13 |
| Teixeira et al. (Brazil, 2017) | 948 | 1,039 | 91.24 [ 89.44, 92.89] | 9.07 |
| Alfaro-Seguro et al. (Costa Rica, 2020) | 46,848 | 53,003 | 88.39 [ 88.11, 88.66] | 9.15 |
| Guimaraes et al. (Brazil, 2021) | 6,253 | 6,737 | 92.82 [ 92.19, 93.42] | 9.13 |
| Ruiz et al. (Argentina, 2021) | 9,909 | 10,762 | 92.07 [ 91.56, 92.58] | 9.14 |
| Heterogeneity: $\tau^2 = 0.03$, $I^2 = 99.68\%$, $H^2 = 313.63$ | | | 88.92 [ 83.52, 93.36] | |
| Test of $\theta_i = \theta_j$: Q(4) = 847.15, p = 0.00 | | | | |
| **Overall** | | | 88.44 [ 85.22, 91.31] | |
| Heterogeneity: $\tau^2 = 0.02$, $I^2 = 99.33\%$, $H^2 = 148.98$ | | | | |
| Test of $\theta_i = \theta_j$: Q(8) = 900.06, p = 0.00 | | | | |
| Test of group differences: $Q_b(1) = 0.13$, p = 0.72 | | | | |

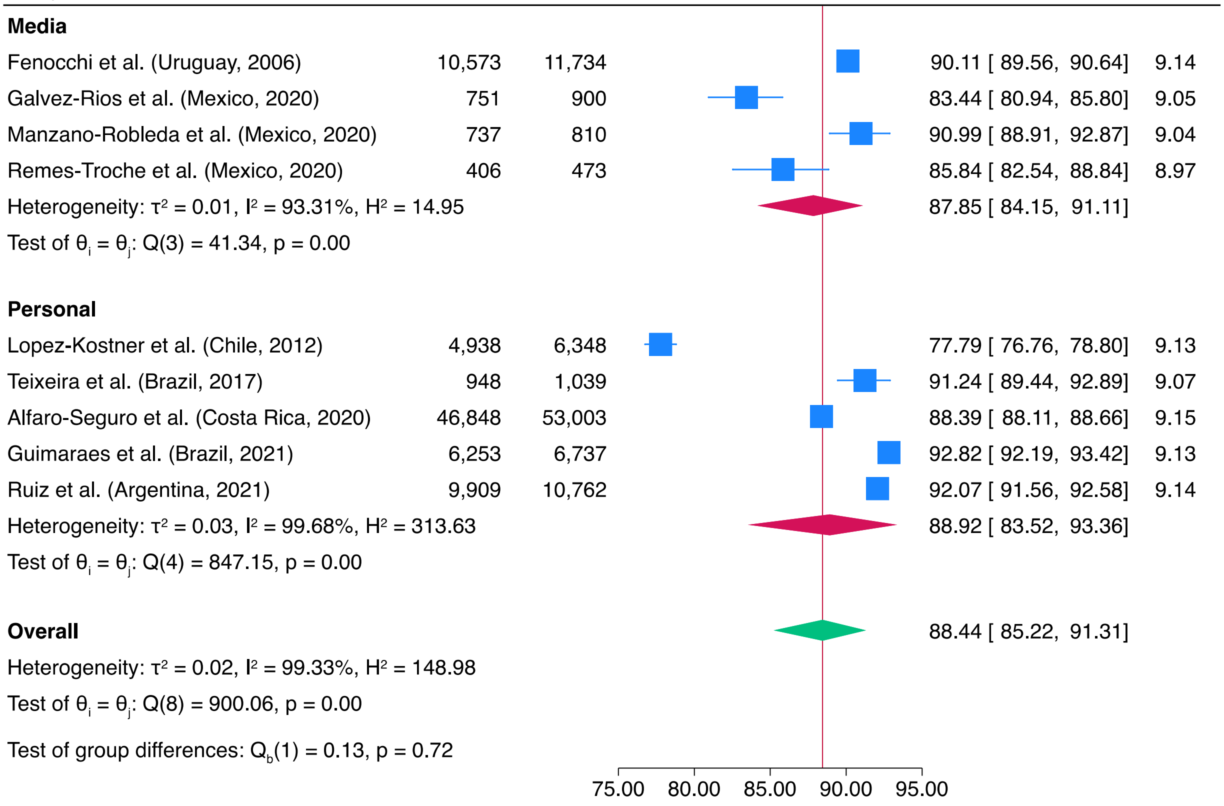


Random-effects REML model
Sorted by: year_pub author

© 2024 Montalvan-Sanchez EE et al. *JAMA Network Open.*

## eFigure 4. Participation Rate (FIT Given/FIT Returned) by Sample Size

| Study | Number of FIT Returned | Total | Prevalence with 95% CI | Weight (%) |
|---|---|---|---|---|
| **Sample <1000** | | | | |
| Galvez-Rios et al. (Mexico, 2020) | 751 | 900 | 83.44 [ 80.94, 85.80] | 9.05 |
| Manzano-Robleda et al. (Mexico, 2020) | 737 | 810 | 90.99 [ 88.91, 92.87] | 9.04 |
| Remes-Troche et al. (Mexico, 2020) | 406 | 473 | 85.84 [ 82.54, 88.84] | 8.97 |
| Fernandez et al. (Argentina, 2021) | 325 | 730 | 44.52 [ 40.93, 48.14] | 9.03 |
| Heterogeneity: $\tau^2$ = 0.23, $I^2$ = 99.39%, $H^2$ = 164.69 | | | 78.04 [ 56.23, 93.78] | |
| Test of $\theta_i = \theta_j$: Q(3) = 506.38, p = 0.00 | | | | |
| **Sample 1000-10,000** | | | | |
| Lopez-Kostner et al. (Chile, 2012) | 4,938 | 6,348 | 77.79 [ 76.76, 78.80] | 9.13 |
| Teixeira et al. (Brazil, 2017) | 948 | 1,039 | 91.24 [ 89.44, 92.89] | 9.07 |
| Guimaraes et al. (Brazil, 2021) | 6,253 | 6,737 | 92.82 [ 92.19, 93.42] | 9.13 |
| Heterogeneity: $\tau^2$ = 0.06, $I^2$ = 99.56%, $H^2$ = 226.86 | | | 87.95 [ 77.88, 95.27] | |
| Test of $\theta_i = \theta_j$: Q(2) = 651.66, p = 0.00 | | | | |
| **Sample >10,000** | | | | |
| Fenocchi et al. (Uruguay, 2006) | 10,573 | 11,734 | 90.11 [ 89.56, 90.64] | 9.14 |
| Alfaro-Seguro et al. (Costa Rica, 2020) | 46,848 | 53,003 | 88.39 [ 88.11, 88.66] | 9.15 |
| Ruiz et al. (Argentina, 2021) | 9,909 | 10,762 | 92.07 [ 91.56, 92.58] | 9.14 |
| Heterogeneity: $\tau^2$ = 0.00, $I^2$ = 98.53%, $H^2$ = 68.14 | | | 90.23 [ 88.03, 92.23] | |
| Test of $\theta_i = \theta_j$: Q(2) = 150.55, p = 0.00 | | | | |
| **Overall** | | | 85.11 [ 76.90, 91.76] | |
| Heterogeneity: $\tau^2$ = 0.11, $I^2$ = 99.87%, $H^2$ = 741.64 | | | | |
| Test of $\theta_i = \theta_j$: Q(9) = 1621.95, p = 0.00 | | | | |
| Test of group differences: $Q_b(2)$ = 2.19, p = 0.33 | | | | |

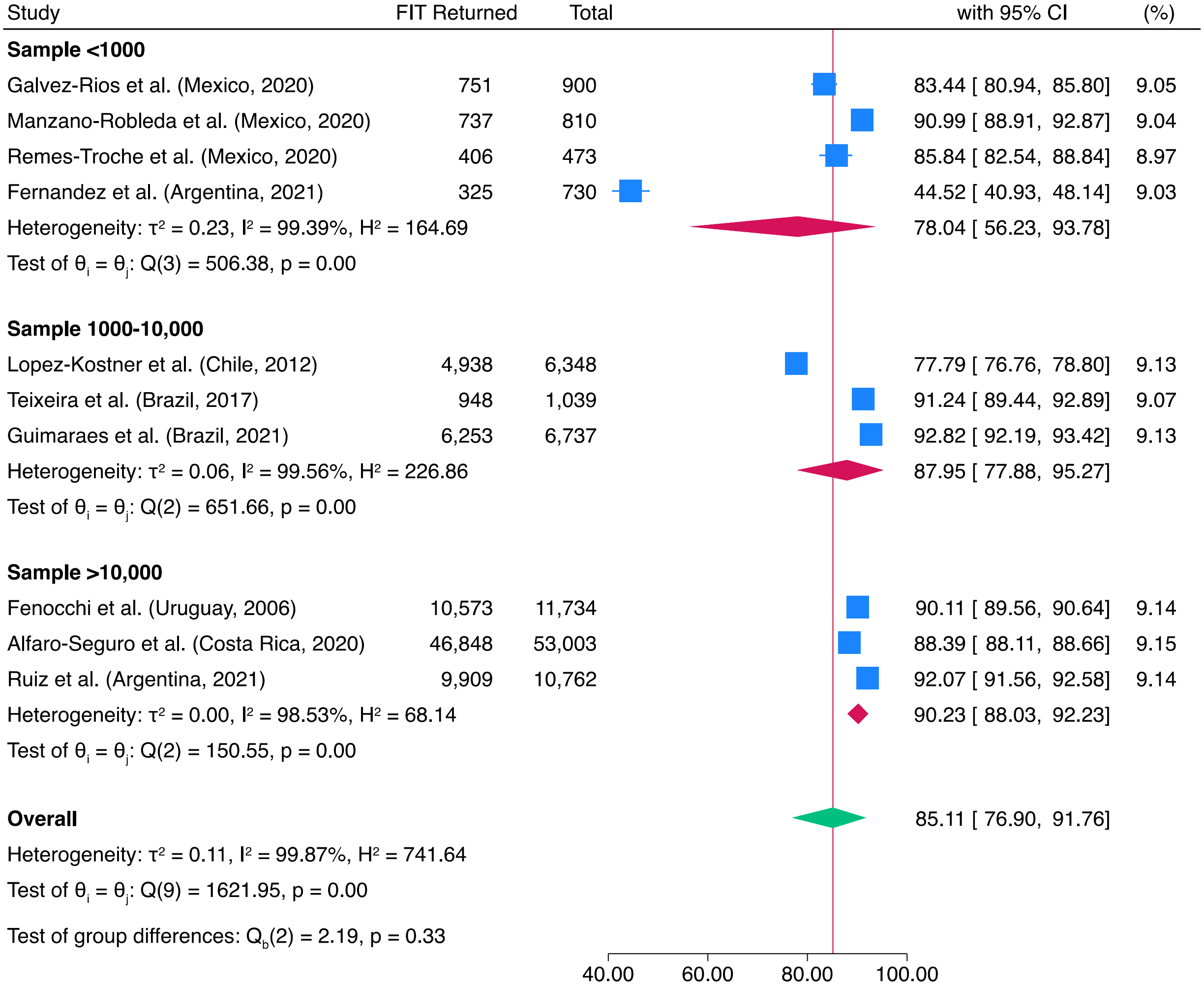


Random-effects REML model
Sorted by: year_pub author

© 2024 Montalvan-Sanchez EE et al. *JAMA Network Open.*

## eFigure 5. Colonoscopy follow-up (colonoscopies performed after a positive FIT test).

| Study | Number of Colonoscopies | FIT Positive | Prevalence with 95% CI | Weight (%) |
|---|---|---|---|---|
| Fenocchi et al. (Uruguay, 2006) | 879 | 1,170 | 75.13 [ 72.61, 77.56] | 7.89 |
| Lopez-Kostner et al. (Chile, 2012) | 279 | 476 | 58.61 [ 54.15, 63.00] | 7.86 |
| Fenocchi et al. (Uruguay, 2015) | 902 | 902 | 100.00 [ 99.81, 100.00] | 7.89 |
| Braga et al. (Brazil, 2017) | 35 | 40 | 87.50 [ 75.17, 96.26] | 7.36 |
| Teixeira et al. (Brazil, 2017) | 73 | 92 | 79.35 [ 70.41, 87.07] | 7.66 |
| Alfaro-Seguro et al. (Costa Rica, 2020) | 3,761 | 3,761 | 100.00 [ 99.95, 100.00] | 7.91 |
| Galvez-Rios et al. (Mexico, 2020) | 40 | 51 | 78.43 [ 65.97, 88.78] | 7.47 |
| Manzano-Robleda et al. (Mexico, 2020) | 58 | 71 | 81.69 [ 71.75, 89.93] | 7.59 |
| Okada et al. (Chile, 2020) | 2,980 | 3,623 | 82.25 [ 80.99, 83.48] | 7.91 |
| Remes-Troche et al. (Mexico, 2020) | 21 | 24 | 87.50 [ 70.72, 98.27] | 7.04 |
| Fernandez et al. (Argentina, 2021) | 36 | 81 | 44.44 [ 33.74, 55.41] | 7.63 |
| Guimaraes et al. (Brazil, 2021) | 659 | 779 | 84.60 [ 81.97, 87.05] | 7.88 |
| Ruiz et al. (Argentina, 2021) | 553 | 2,209 | 25.03 [ 23.25, 26.86] | 7.90 |
| **Overall** | | | 79.56 [ 65.42, 90.78] | |

Heterogeneity: $\tau^2 = 0.33$, $I^2 = 99.66\%$, $H^2 = 290.08$

Test of $\theta_i = \theta_j$: $Q(12) = 6861.15$, $p = 0.00$

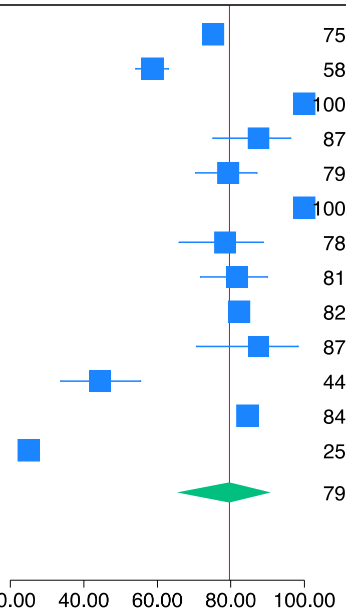


Random-effects REML model
Sorted by: year_pub author

© 2024 Montalvan-Sanchez EE et al. *JAMA Network Open.*

**eFigure 6. Outcome ADR (Adenoma Detection Rate) per positive FIT test in FIT Based programs.**

| Study | Number of Adenomas | Total | Prevalence with 95% CI | Weight (%) |
|---|---|---|---|---|
| **50 ng/ml or Less** | | | | |
| Teixeira et al. (Brazil, 2017) | 43 | 92 | 46.74 [ 36.60, 57.01] | 8.27 |
| Manzano-Robleda et al. (Mexico, 2020) | 36 | 71 | 50.70 [ 39.04, 62.33] | 8.09 |
| Fernandez et al. (Argentina, 2021) | 5 | 81 | 6.17 [ 1.78, 12.64] | 8.19 |
| Guimaraes et al. (Brazil, 2021) | 429 | 779 | 55.07 [ 51.56, 58.55] | 8.84 |
| Ruiz et al. (Argentina, 2021) | 121 | 2,209 | 5.48 [ 4.57, 6.47] | 8.89 |
| Heterogeneity: $\tau^2$ = 0.35, $I^2$ = 99.27%, $H^2$ = 136.76 | | | 29.44 [ 9.08, 55.40] | |
| Test of $\theta_i = \theta_j$: Q(4) = 924.20, p = 0.00 | | | | |
| **100 ng/ml** | | | | |
| Fenocchi et al. (Uruguay, 2006) | 330 | 1,170 | 28.21 [ 25.66, 30.82] | 8.87 |
| Lopez-Kostner et al. (Chile, 2012) | 136 | 476 | 28.57 [ 24.60, 32.72] | 8.79 |
| Galvez-Rios et al. (Mexico, 2020) | 11 | 51 | 21.57 [ 11.22, 34.03] | 7.81 |
| Okada et al. (Chile, 2020) | 1,396 | 3,623 | 38.53 [ 36.95, 40.12] | 8.91 |
| Remes-Troche et al. (Mexico, 2020) | 6 | 24 | 25.00 [ 9.38, 44.53] | 6.87 |
| Heterogeneity: $\tau^2$ = 0.01, $I^2$ = 90.53%, $H^2$ = 10.56 | | | 29.90 [ 24.16, 35.96] | |
| Test of $\theta_i = \theta_j$: Q(4) = 58.56, p = 0.00 | | | | |
| **Not reported** | | | | |
| Braga et al. (Brazil, 2017) | 12 | 40 | 30.00 [ 16.65, 45.24] | 7.56 |
| Alfaro-Seguro et al. (Costa Rica, 2020) | 852 | 3,761 | 22.65 [ 21.33, 24.01] | 8.91 |
| Heterogeneity: $\tau^2$ = 0.00, $I^2$ = 20.76%, $H^2$ = 1.26 | | | 23.17 [ 18.63, 28.03] | |
| Test of $\theta_i = \theta_j$: Q(1) = 1.26, p = 0.26 | | | | |
| **Overall** | | | 28.45 [ 19.14, 38.77] | |
| Heterogeneity: $\tau^2$ = 0.14, $I^2$ = 99.17%, $H^2$ = 120.24 | | | | |
| Test of $\theta_i = \theta_j$: Q(11) = 1421.20, p = 0.00 | | | | |
| Test of group differences: $Q_b(2)$ = 3.15, p = 0.21 | | | | |

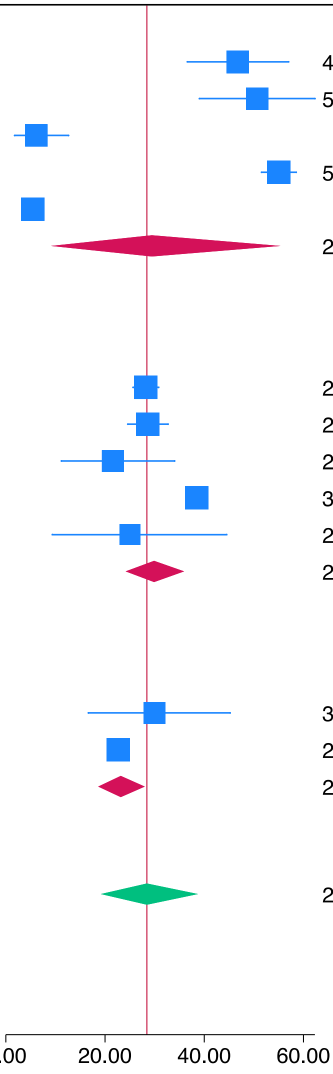


Random-effects REML model
Sorted by: year_pub author

Note: Test of group differences p-value=0.97 excluding “Not reported” category.

© 2024 Montalvan-Sanchez EE et al. *JAMA Network Open.*

## eFigure 7. Outcome AADR (Advanced Adenomas Detection Rate) per positive FIT test in FIT Based programs.

| Study | Number of Adv. Adenomas | Total | Prevalence with 95% CI | Weight (%) |
|---|---|---|---|---|
| **50 ng/ml or Less** | | | | |
| Teixeira et al. (Brazil, 2017) | 7 | 92 | 7.61 [ 2.93, 14.05] | 10.62 |
| Manzano-Robleda et al. (Mexico, 2020) | 20 | 71 | 28.17 [ 18.24, 39.26] | 10.30 |
| Fernandez et al. (Argentina, 2021) | 1 | 81 | 1.23 [ 0.95, 5.23] | 10.47 |
| Guimaraes et al. (Brazil, 2021) | 143 | 779 | 18.36 [ 15.71, 21.16] | 11.73 |
| Ruiz et al. (Argentina, 2021) | 45 | 2,209 | 2.04 [ 1.49, 2.67] | 11.84 |
| Heterogeneity: $\tau^2$ = 0.13, $I^2$ = 98.04%, $H^2$ = 50.94 | | | 9.16 [ 1.88, 20.74] | |
| Test of $\theta_i = \theta_j$: Q(4) = 243.18, p = 0.00 | | | | |
| **100 ng/ml** | | | | |
| Fenocchi et al. (Uruguay, 2006) | 131 | 1,170 | 11.20 [ 9.45, 13.07] | 11.78 |
| Lopez-Kostner et al. (Chile, 2012) | 75 | 476 | 15.76 [ 12.61, 19.17] | 11.62 |
| Galvez-Rios et al. (Mexico, 2020) | 3 | 51 | 5.88 [ 0.76, 14.37] | 9.78 |
| Heterogeneity: $\tau^2$ = 0.01, $I^2$ = 78.15%, $H^2$ = 4.58 | | | 11.85 [ 7.84, 16.54] | |
| Test of $\theta_i = \theta_j$: Q(2) = 8.14, p = 0.02 | | | | |
| **Not reported** | | | | |
| Alfaro-Seguro et al. (Costa Rica, 2020) | 43 | 3,761 | 1.14 [ 0.83, 1.51] | 11.86 |
| Heterogeneity: $\tau^2$ = 0.00, $I^2$ = .%, $H^2$ = . | | | 1.14 [ 0.83, 1.51] | |
| Test of $\theta_i = \theta_j$: Q(0) = 0.00, p = . | | | | |
| **Overall** | | | 8.33 [ 3.50, 14.87] | |
| Heterogeneity: $\tau^2$ = 0.09, $I^2$ = 98.60%, $H^2$ = 71.32 | | | | |
| Test of $\theta_i = \theta_j$: Q(8) = 560.40, p = 0.00 | | | | |
| Test of group differences: $Q_b(2)$ = 56.03, p = 0.00 | | | | |

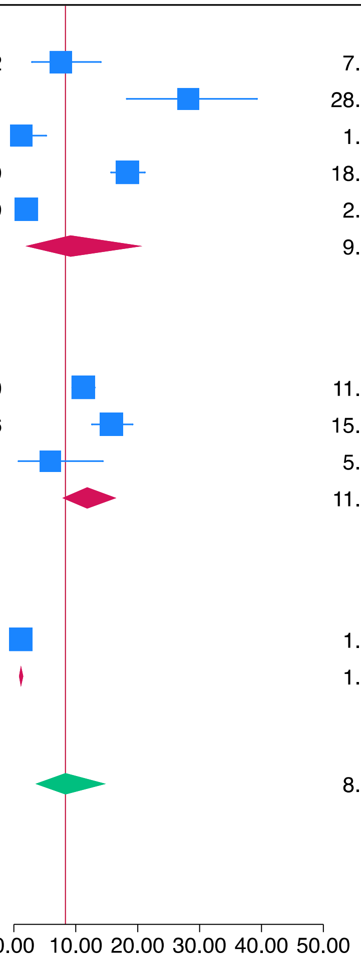


Random-effects REML model
Sorted by: year_pub author

Note: Test of group differences p-value=0.02 excluding “Not reported” category.

© 2024 Montalvan-Sanchez EE et al. *JAMA Network Open.*

## eFigure 8. Outcome CRCDR (Colorectal Cancer Detection Rate) per positive FIT test in FIT Based programs.

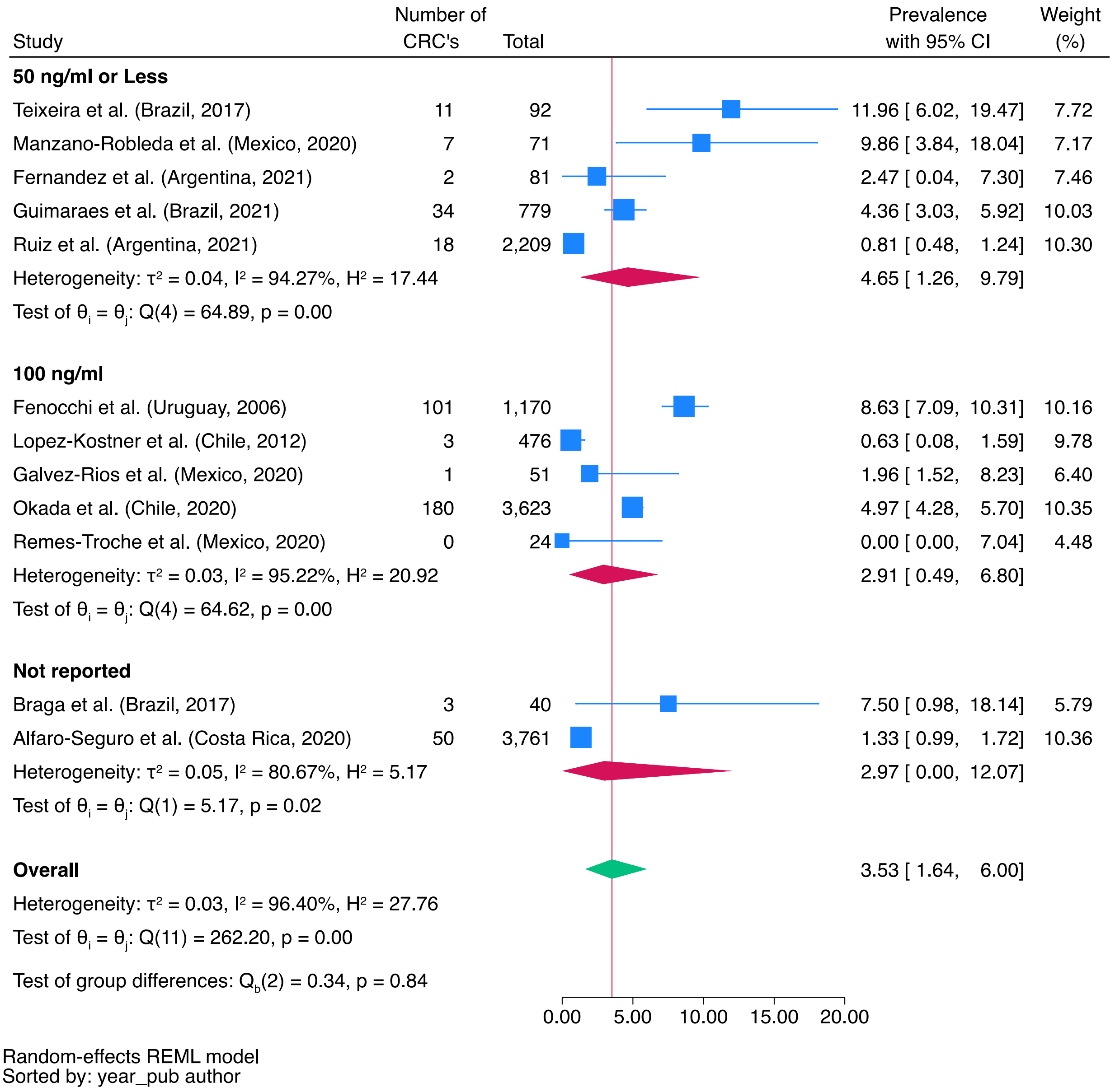


Note: Test of group differences p-value=0.86 excluding “Not reported” category.

© 2024 Montalvan-Sanchez EE et al. *JAMA Network Open.*

## eFigure 9. Yield of Colonoscopy: AADR (Advanced Adenomas Detection Rate). FIT programs

| Study | Number of Adv. Adenomas | Total | Prevalence with 95% CI | Weight (%) |
|---|---|---|---|---|
| **FIT-positive, average risk but family history NR or effort not made to exclude** | | | | |
| Fenocchi et al. (Uruguay, 2015) | 81 | 902 | 8.98 [ 7.20, 10.94] | 10.82 |
| Teixeira et al. (Brazil, 2017) | 18 | 73 | 24.66 [ 15.37, 35.26] | 9.46 |
| Galvez-Rios et al. (Mexico, 2020) | 3 | 40 | 7.50 [ 0.98, 18.14] | 8.52 |
| Guimaraes et al. (Brazil, 2021) | 143 | 659 | 21.70 [ 18.63, 24.93] | 10.77 |
| Ruiz et al. (Argentina, 2021) | 45 | 553 | 8.14 [ 5.99, 10.57] | 10.73 |
| Heterogeneity: $\tau^2 = 0.04$, $I^2 = 94.43\%$, $H^2 = 17.96$ | | | 13.42 [ 7.29, 21.00] | |
| Test of $\theta_i = \theta_j$: Q(4) = 71.29, p = 0.00 | | | | |
| **FIT-positive, average risk and family history excluded** | | | | |
| Fenocchi et al. (Uruguay, 2006) | 232 | 879 | 26.39 [ 23.53, 29.36] | 10.81 |
| Lopez-Kostner et al. (Chile, 2012) | 21 | 279 | 7.53 [ 4.69, 10.94] | 10.52 |
| Alfaro-Seguro et al. (Costa Rica, 2020) | 82 | 3,761 | 2.18 [ 1.74, 2.67] | 10.92 |
| Manzano-Robleda et al. (Mexico, 2020) | 20 | 58 | 34.48 [ 22.72, 47.26] | 9.14 |
| Fernandez et al. (Argentina, 2021) | 3 | 36 | 8.33 [ 1.10, 20.05] | 8.31 |
| Heterogeneity: $\tau^2 = 0.15$, $I^2 = 98.70\%$, $H^2 = 77.19$ | | | 13.37 [ 3.67, 27.53] | |
| Test of $\theta_i = \theta_j$: Q(4) = 478.69, p = 0.00 | | | | |
| **Overall** | | | 13.34 [ 7.44, 20.57] | |
| Heterogeneity: $\tau^2 = 0.09$, $I^2 = 97.94\%$, $H^2 = 48.50$ | | | | |
| Test of $\theta_i = \theta_j$: Q(9) = 654.96, p = 0.00 | | | | |
| Test of group differences: $Q_b(1) = 0.00$, p = 1.00 | | | | |

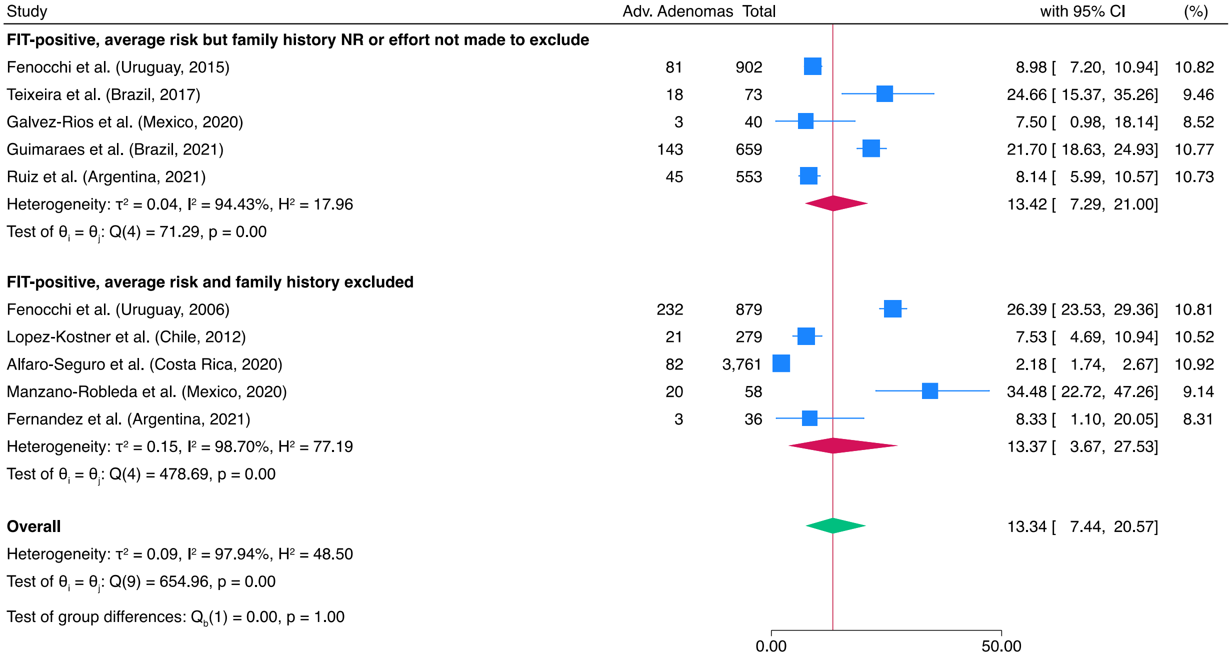


Random-effects REML model
Sorted by: year_pub author

© 2024 Montalvan-Sanchez EE et al. *JAMA Network Open.*

## eFigure 10. Yield of Colonoscopy: AADR (Advanced Adenomas Detection Rate). Direct-to-colonoscopy programs

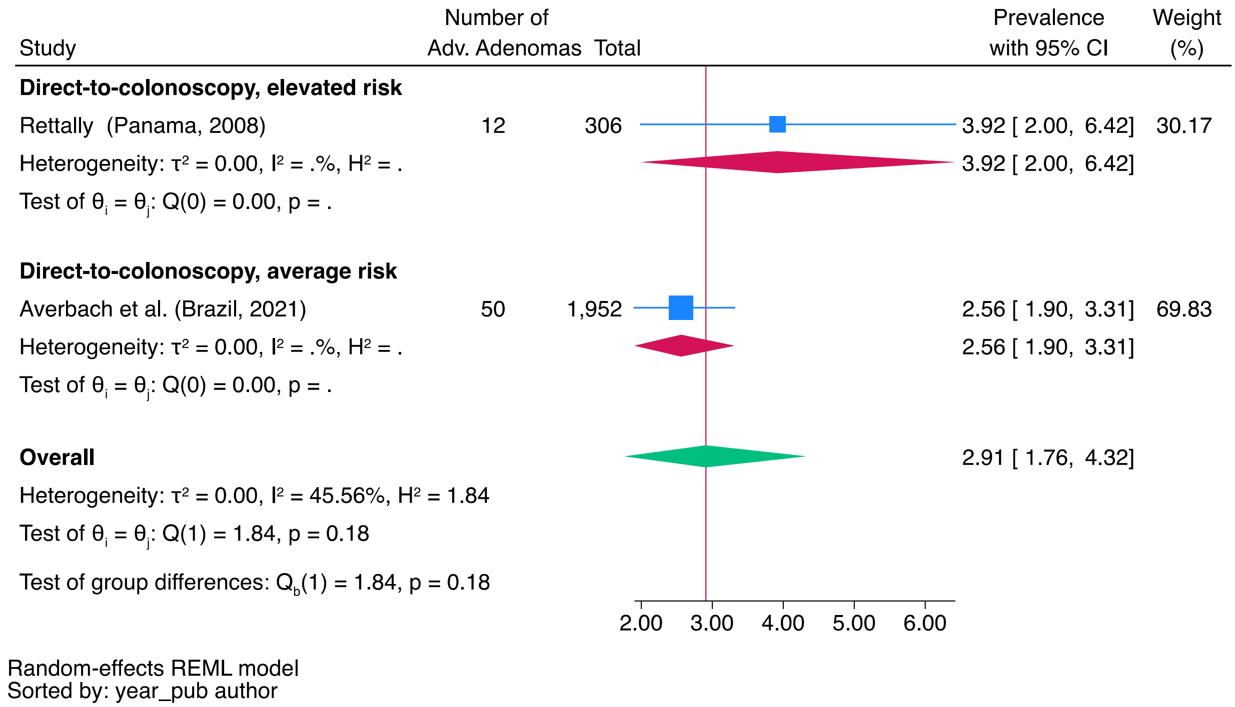


© 2024 Montalvan-Sanchez EE et al. *JAMA Network Open.*

## eFigure 11. Yield of Colonoscopy: CRC-DR (Adenocarcinoma Detection Rate) by type of program

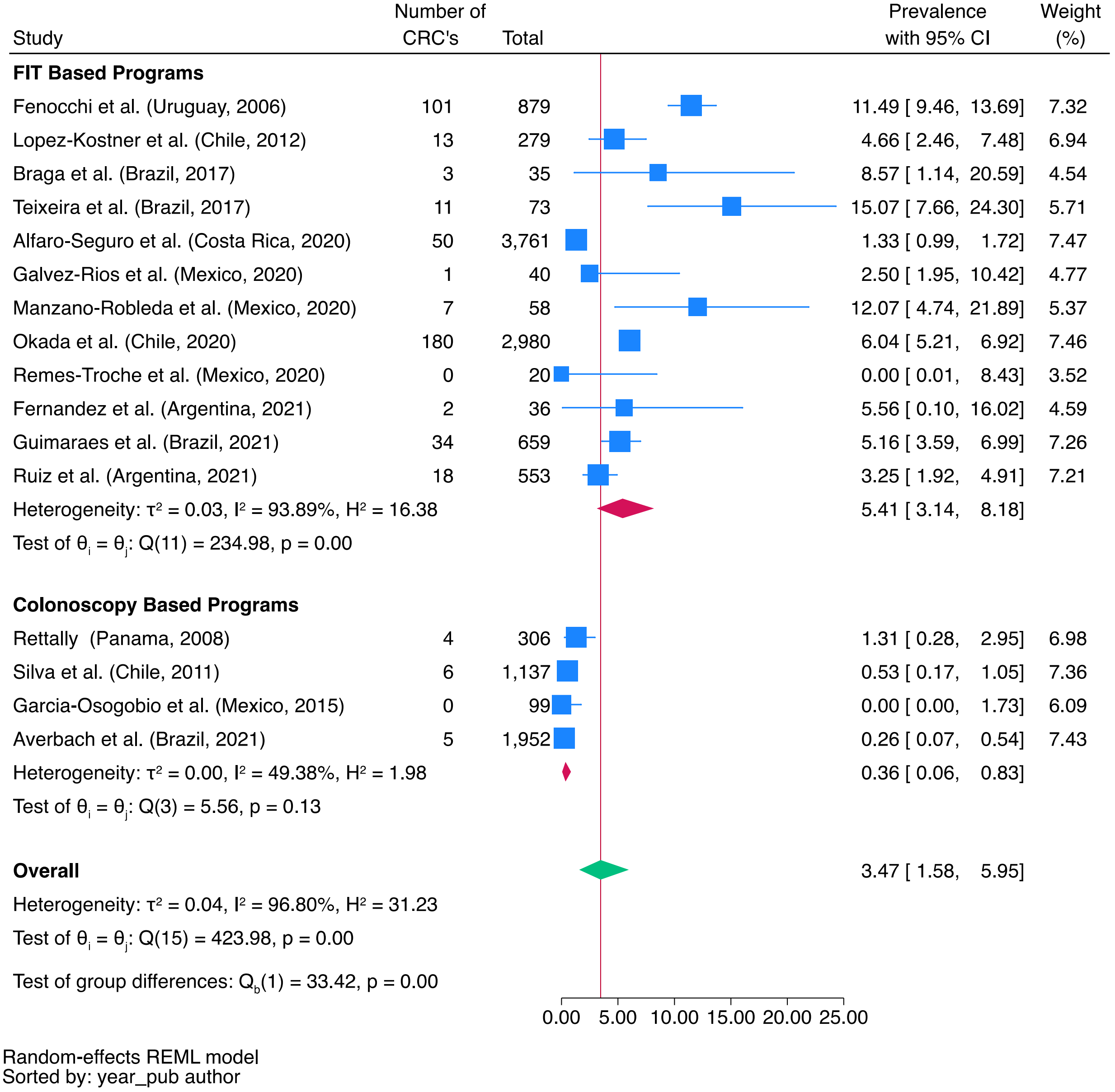


© 2024 Montalvan-Sanchez EE et al. *JAMA Network Open.*

## eFigure 12. Colonoscopy Quality Indicators: Cecal Intubation Rate

| Study | Number of Cecal Intubations | Total | Prevalence with 95% CI | Weight (%) |
|---|---|---|---|---|
| **FIT Based Programs** | | | | |
| Fenocchi et al. (Uruguay, 2006) | 763 | 879 | 86.80 [ 84.48, 88.96] | 9.30 |
| Lopez-Kostner et al. (Chile, 2012) | 245 | 279 | 87.81 [ 83.70, 91.41] | 8.80 |
| Teixeira et al. (Brazil, 2017) | 73 | 73 | 100.00 [ 97.66, 100.00] | 7.20 |
| Alfaro-Seguro et al. (Costa Rica, 2020) | 3,460 | 3,761 | 92.00 [ 91.11, 92.84] | 9.49 |
| Manzano-Robleda et al. (Mexico, 2020) | 56 | 58 | 95.86 [ 88.83, 99.75] | 6.78 |
| Okada et al. (Chile, 2020) | 2,851 | 2,980 | 95.67 [ 94.91, 96.37] | 9.47 |
| Remes-Troche et al. (Mexico, 2020) | 20 | 20 | 100.00 [ 91.57, 99.99] | 4.41 |
| Guimaraes et al. (Brazil, 2021) | 654 | 659 | 99.24 [ 98.41, 99.79] | 9.22 |
| Heterogeneity: $\tau^2 = 0.05$, $I^2 = 97.77\%$, $H^2 = 44.93$ | | | 95.55 [ 91.20, 98.56] | |
| Test of $\theta_i = \theta_j$: Q(7) = 184.09, p = 0.00 | | | | |
| **Colonoscopy Based Programs** | | | | |
| Rettally (Panama, 2008) | 300 | 306 | 98.04 [ 96.13, 99.35] | 8.86 |
| Silva et al. (Chile, 2011) | 1,092 | 1,137 | 96.04 [ 94.83, 97.10] | 9.35 |
| Garcia-Osogobio et al. (Mexico, 2015) | 87 | 99 | 87.88 [ 80.63, 93.67] | 7.70 |
| Averbach et al. (Brazil, 2021) | 1,914 | 1,952 | 98.05 [ 97.39, 98.62] | 9.43 |
| Heterogeneity: $\tau^2 = 0.03$, $I^2 = 95.34\%$, $H^2 = 21.46$ | | | 96.05 [ 91.73, 98.88] | |
| Test of $\theta_i = \theta_j$: Q(3) = 26.26, p = 0.00 | | | | |
| **Overall** | | | 95.67 [ 92.69, 97.95] | |
| Heterogeneity: $\tau^2 = 0.04$, $I^2 = 97.38\%$, $H^2 = 38.17$ | | | | |
| Test of $\theta_i = \theta_j$: Q(11) = 285.43, p = 0.00 | | | | |
| Test of group differences: $Q_b(1) = 0.10$, p = 0.76 | | | | |

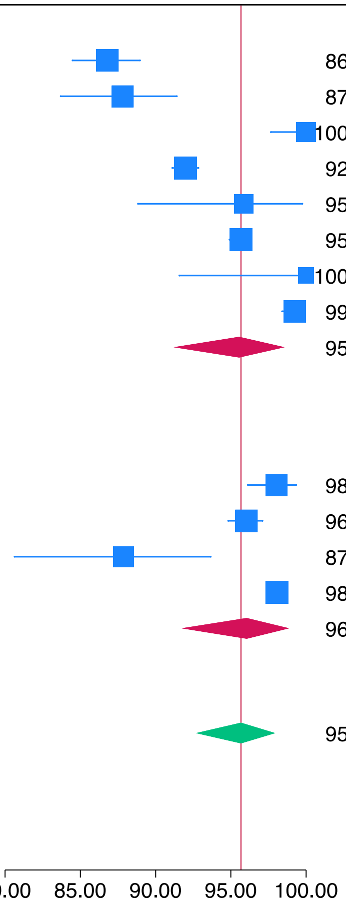


Random-effects REML model
Sorted by: year_pub author

© 2024 Montalvan-Sanchez EE et al. *JAMA Network Open.*

## eFigure 13. Colonoscopy Quality Indicators: Mean Withdrawal Time from cecum (mean, minutes)

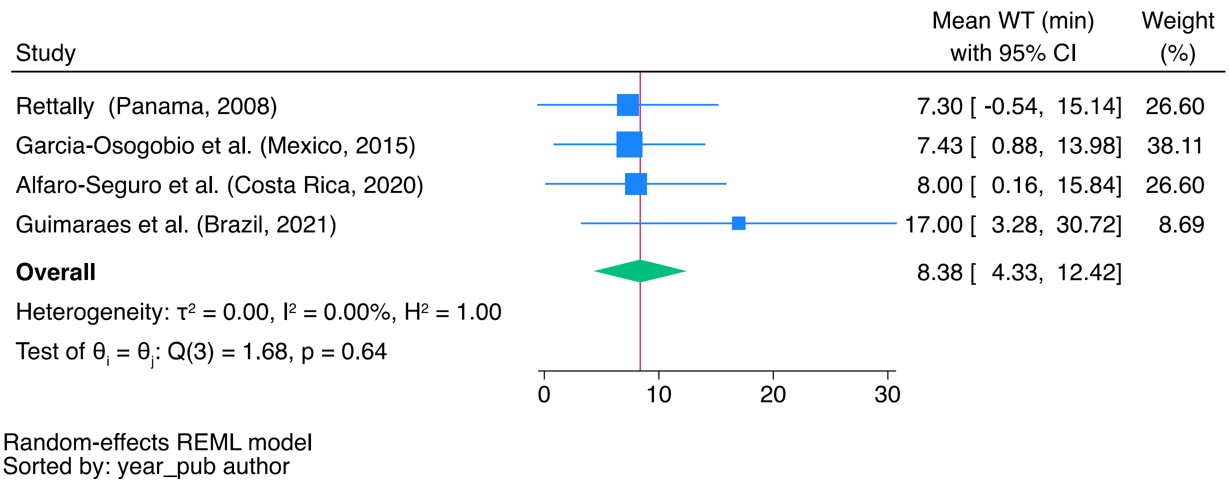


© 2024 Montalvan-Sanchez EE et al. *JAMA Network Open.*

## eFigure 14. Colonoscopy Quality Indicators: Adequate Bowel Preparation

| Study | Number of Adequate Prep. | Total | Prevalence with 95% CI | Weight (%) |
|---|---|---|---|---|
| **FIT Based Programs** | | | | |
| Alfaro-Seguro et al. (Costa Rica, 2020) | 3,448 | 3,761 | 91.68 [ 90.77, 92.54] | 26.19 |
| Manzano-Robleda et al. (Mexico, 2020) | 48 | 58 | 83.52 [ 72.73, 92.11] | 21.88 |
| Guimaraes et al. (Brazil, 2021) | 641 | 659 | 97.27 [ 95.87, 98.39] | 25.81 |
| Heterogeneity: $\tau^2 = 0.05$, $I^2 = 97.06\%$, $H^2 = 34.03$ | | | 92.34 [ 83.34, 98.13] | |
| Test of $\theta_i = \theta_j$: Q(2) = 40.16, p = 0.00 | | | | |
| **Colonoscopy Based Programs** | | | | |
| Averbach et al. (Brazil, 2021) | 1,944 | 1,952 | 99.59 [ 99.25, 99.83] | 26.11 |
| Heterogeneity: $\tau^2 = 0.00$, $I^2 = .\%$, $H^2 = .$ | | | 99.59 [ 99.25, 99.83] | |
| Test of $\theta_i = \theta_j$: Q(0) = 0.00, p = . | | | | |
| **Overall** | | | 95.00 [ 86.62, 99.52] | |
| Heterogeneity: $\tau^2 = 0.09$, $I^2 = 99.02\%$, $H^2 = 102.50$ | | | | |
| Test of $\theta_i = \theta_j$: Q(3) = 280.56, p = 0.00 | | | | |
| Test of group differences: $Q_b(1) = 9.48$, p = 0.00 | | | | |

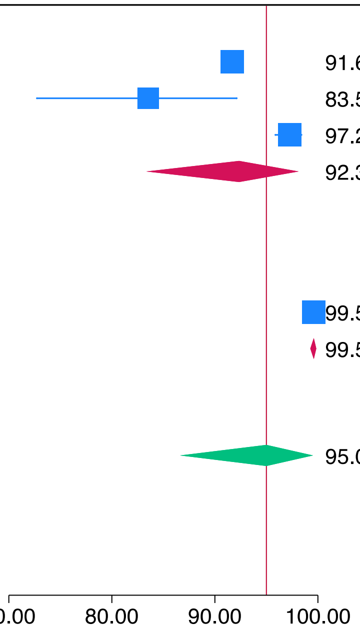


Random-effects REML model
Sorted by: year_pub author

© 2024 Montalvan-Sanchez EE et al. *JAMA Network Open.*

## eFigure 15. Heterogeneity and Publication bias (small study effects) for FIT Based Studies

| Heterogeneity | Publication Bias (small-study effects) |
|---|---|

### Participation Rates

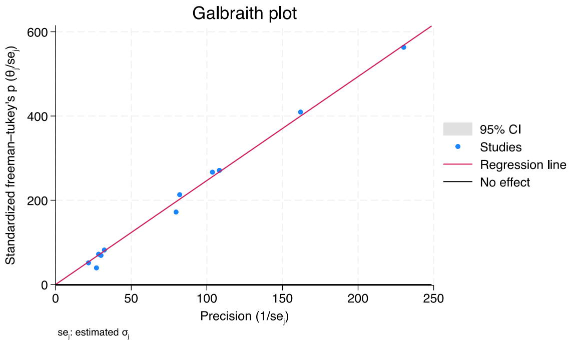


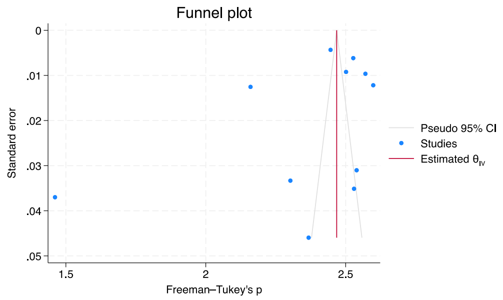


**Egger p-value=0.2010**

### FIT positivity Rates

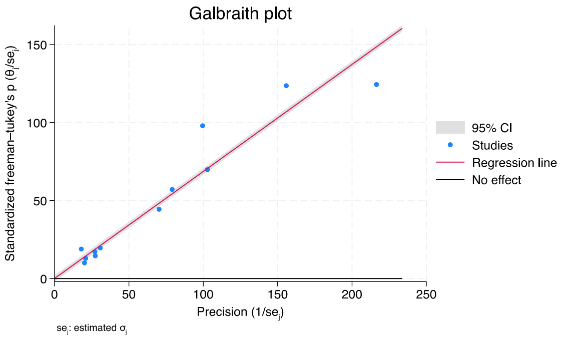


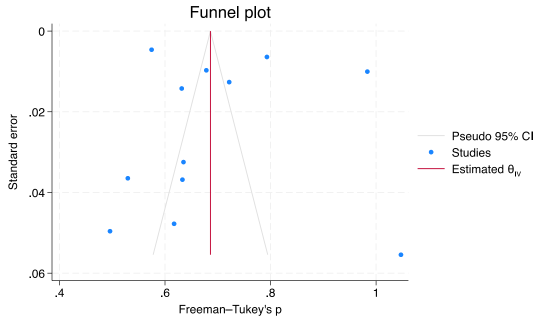


**Egger p-value=0.8101**

© 2024 Montalvan-Sanchez EE et al. *JAMA Network Open.*

## eFigure 16. Heterogeneity and Publication bias (small study effects) for outcomes per positive FIT test in FIT based programs

| Heterogeneity | Publication Bias (small-study effects) |
|---|---|
| **Outcome per positive FIT test in FIT Based programs** | |
| **ADR (Adenoma Detection Rate).** | |

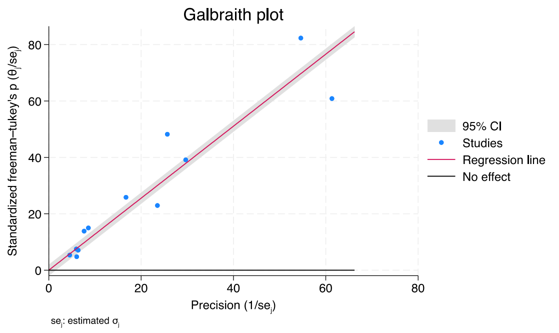


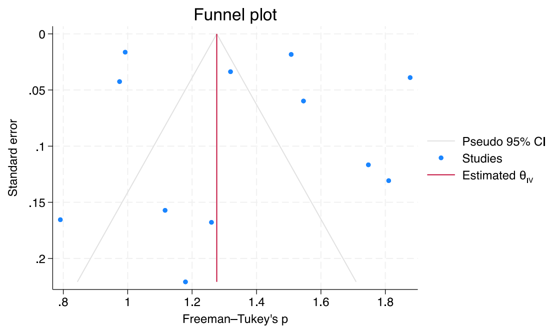


**Egger p-value=0.5886**

**AADR (Advanced Adenomas Detection Rate).**

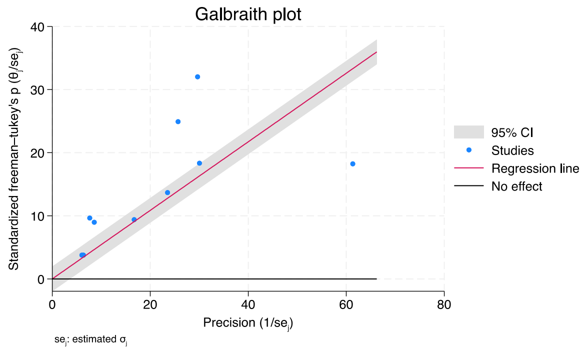


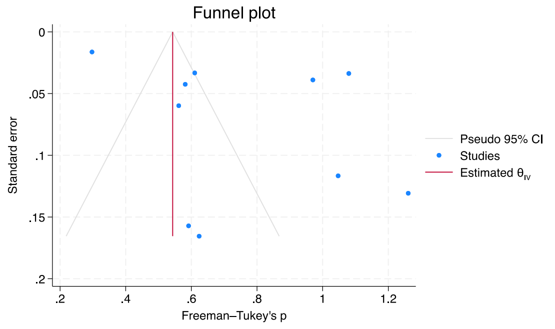


**Egger p-value=0.4609**

**CRC Detection Rates**

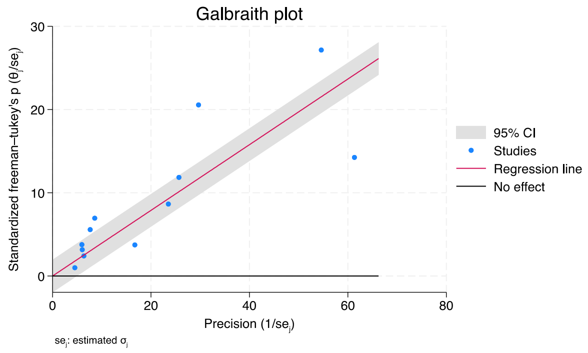


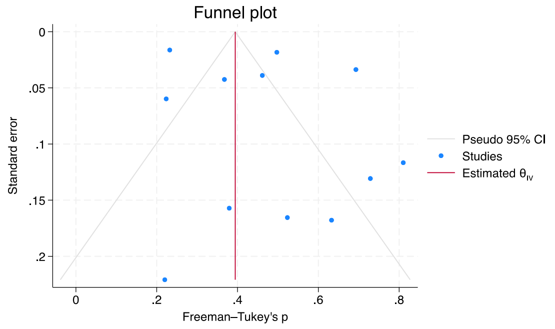


**Egger p-value=0.5318**

© 2024 Montalvan-Sanchez EE et al. *JAMA Network Open.*

## eFigure 17. Heterogeneity and Publication bias (small study effects) for for outcomes in Direct-to-Colonoscopy programs

| Heterogeneity | Publication Bias (small-study effects) |
|---|---|

### Colonoscopy Indicators

#### ADR (Adenoma Detection Rate).

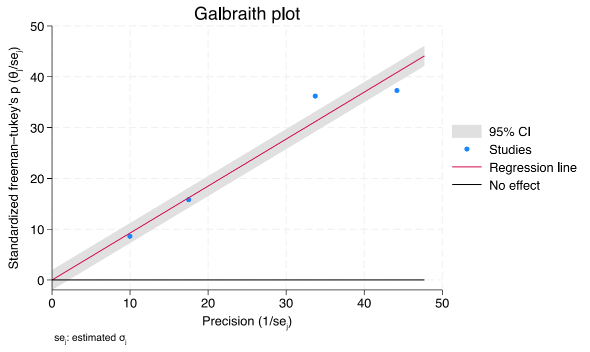


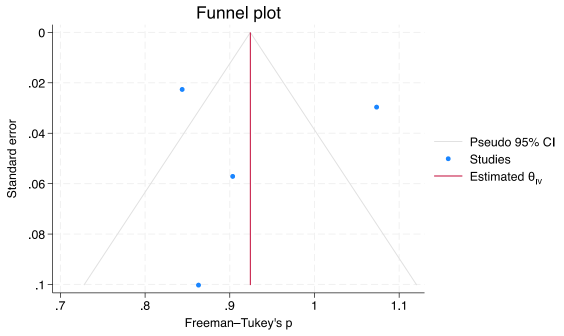


**Egger p-value=0.6699**

#### AADR (Advanced Adenomas Detection Rate).

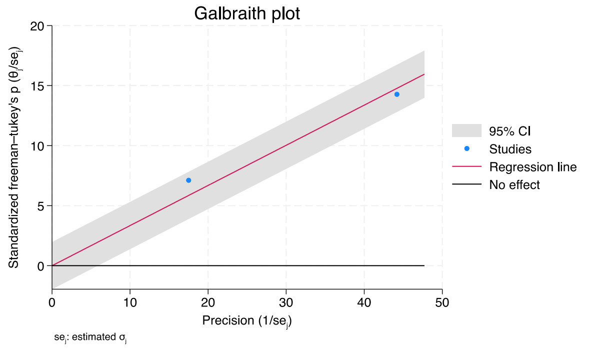


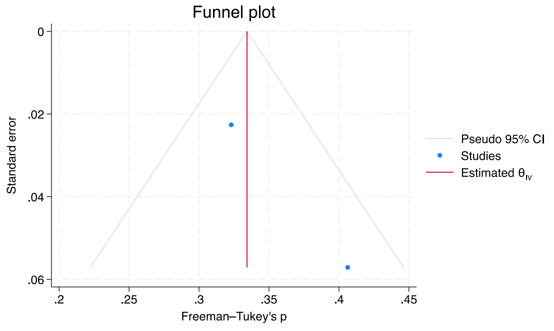


**Egger p-value=.**

### CRC Detection Rates

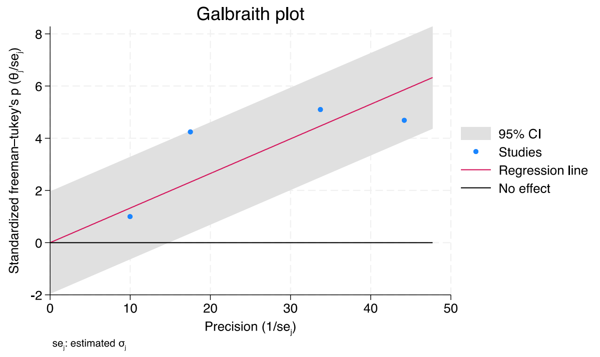


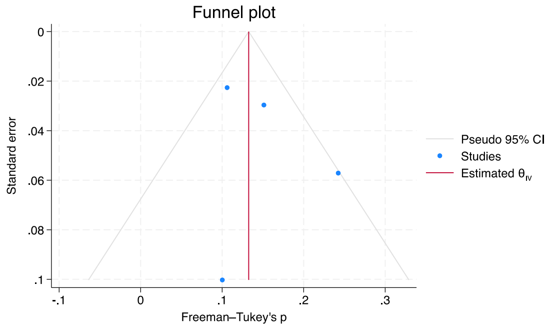


**Egger p-value=0.4836**

© 2024 Montalvan-Sanchez EE et al. *JAMA Network Open.*

# eFigure 18. Heterogeneity and Publication bias (small study effects) for Colonoscopy quality indicators

| Heterogeneity | Publication Bias (small-study effects) |
|---|---|

## Colonoscopy Quality Indicators

### Cecal Intubation Rate

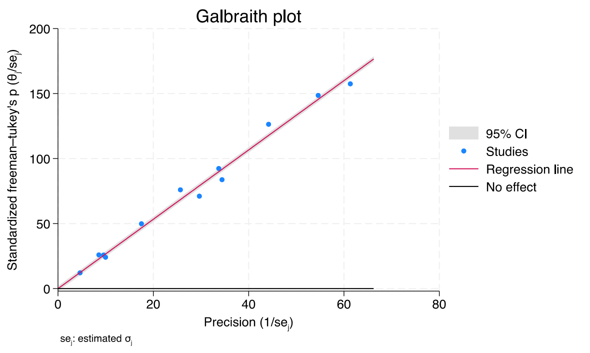


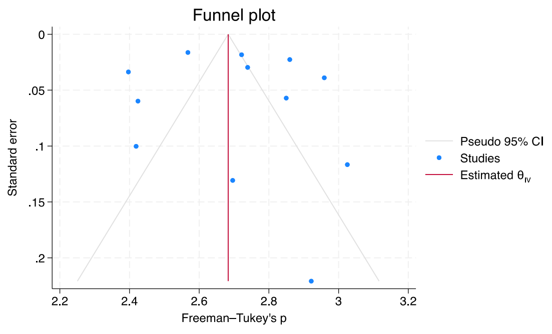


Egger p-value=0.5315

### Mean Withdrawal Time

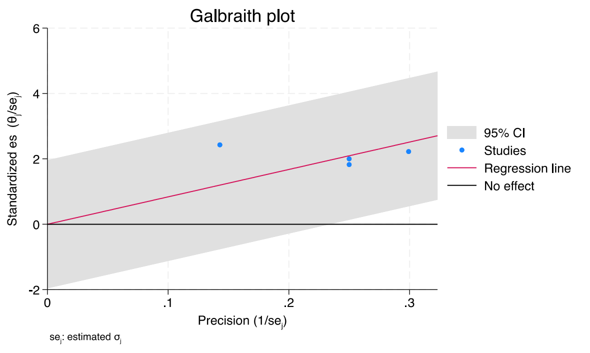


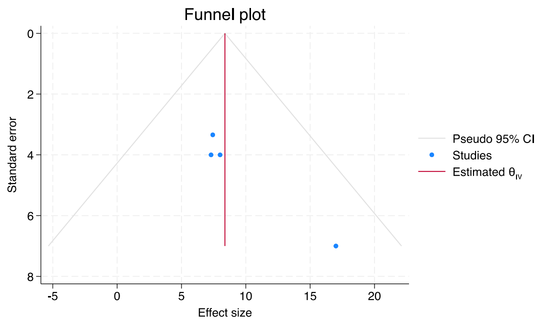


Egger p-value=0.2158

### Adequate Bowel Preparation

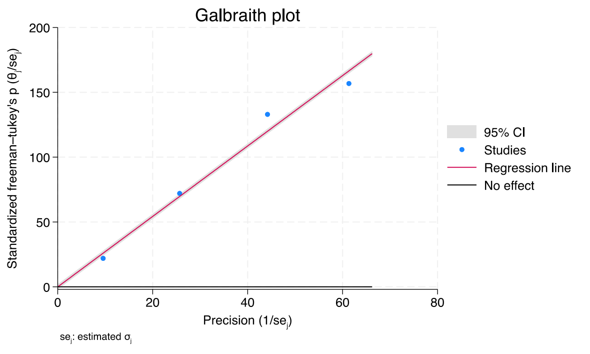


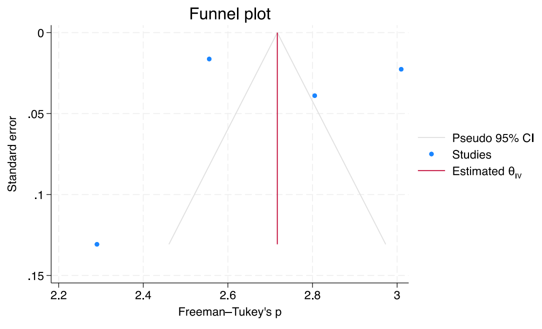


Egger p-value=0.1434

© 2024 Montalvan-Sanchez EE et al. *JAMA Network Open.*

## eFigure 19. ADR (Adenomas Detection Rate) FIT programs by risk. Leave-one-out analysis.

| Study | Number of Adenomas | Total | Prevalence with 95% CI | Weight (%) |
|---|---|---|---|---|
| **FIT-positive, elevated risk** | | | | |
| Braga et al. (Brazil, 2017) | 12 | 35 | 34.29 [ 19.32, 50.94] | 8.51 |
| Okada et al. (Chile, 2020) | 1,396 | 2,980 | 46.85 [ 45.06, 48.64] | 11.58 |
| Heterogeneity: $\tau^2 = 0.02$, $I^2 = 53.49\%$, $H^2 = 2.15$ | | | 43.39 [ 32.76, 54.33] | |
| Test of $\theta_i = \theta_j$: Q(1) = 2.15, p = 0.14 | | | | |
| **FIT-positive, average risk but family history NR or effort not made to exclude** | | | | |
| Teixeira et al. (Brazil, 2017) | 43 | 73 | 58.90 [ 47.37, 69.98] | 9.88 |
| Galvez-Rios et al. (Mexico, 2020) | 11 | 40 | 27.50 [ 14.61, 42.51] | 8.80 |
| Ruiz et al. (Argentina, 2021) | 121 | 553 | 21.88 [ 18.53, 25.43] | 11.36 |
| Heterogeneity: $\tau^2 = 0.16$, $I^2 = 94.08\%$, $H^2 = 16.89$ | | | 35.24 [ 14.85, 58.86] | |
| Test of $\theta_i = \theta_j$: Q(2) = 38.83, p = 0.00 | | | | |
| **FIT-positive, average risk and family history excluded** | | | | |
| Fenocchi et al. (Uruguay, 2006) | 330 | 879 | 37.54 [ 34.37, 40.77] | 11.46 |
| Lopez-Kostner et al. (Chile, 2012) | 136 | 279 | 48.75 [ 42.89, 54.62] | 11.11 |
| Alfaro-Seguro et al. (Costa Rica, 2020) | 852 | 3,761 | 22.65 [ 21.33, 24.01] | 11.59 |
| Remes-Troche et al. (Mexico, 2020) | 6 | 20 | 30.00 [ 11.56, 52.18] | 7.12 |
| Fernandez et al. (Argentina, 2021) | 5 | 36 | 13.89 [ 4.18, 27.40] | 8.58 |
| Heterogeneity: $\tau^2 = 0.08$, $I^2 = 97.38\%$, $H^2 = 38.17$ | | | 30.79 [ 19.31, 43.57] | |
| Test of $\theta_i = \theta_j$: Q(4) = 143.20, p = 0.00 | | | | |
| **Overall** | | | 34.16 [ 25.54, 43.32] | |
| Heterogeneity: $\tau^2 = 0.08$, $I^2 = 98.04\%$, $H^2 = 51.14$ | | | | |
| Test of $\theta_i = \theta_j$: Q(9) = 540.60, p = 0.00 | | | | |
| Test of group differences: $Q_b(2) = 2.27$, p = 0.32 | | | | |

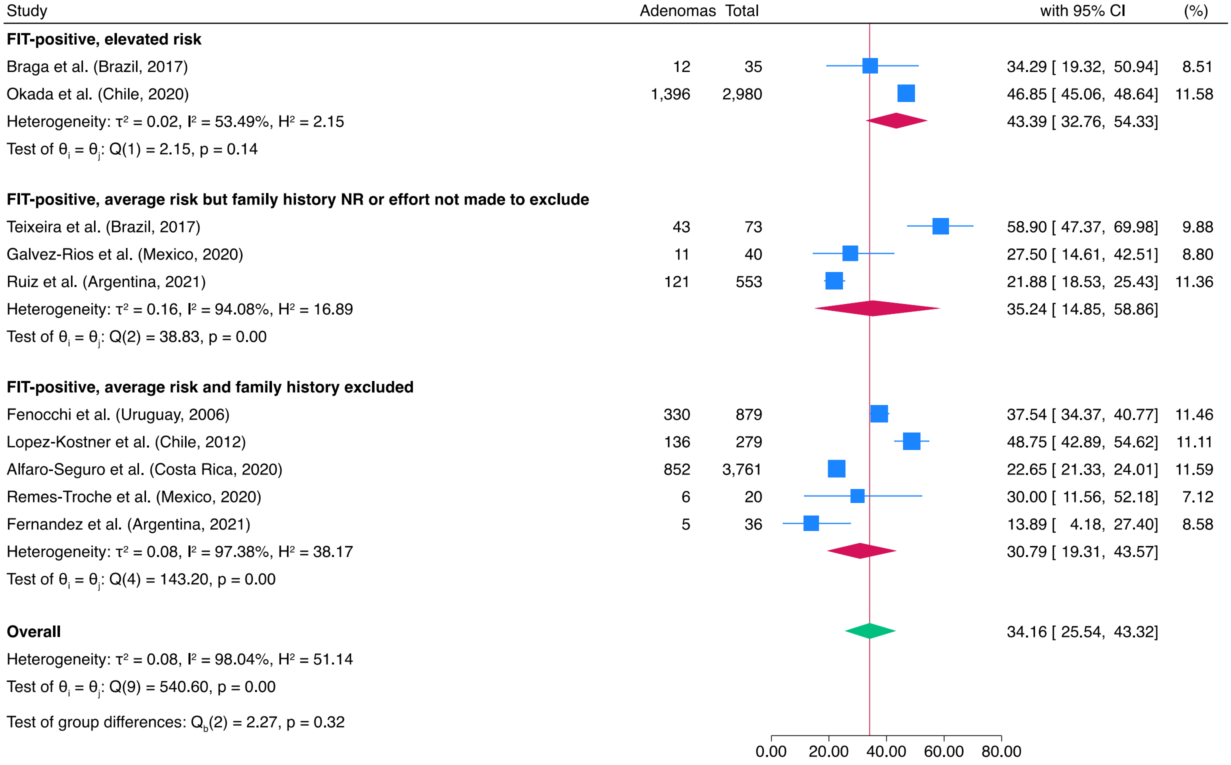


Random-effects REML model
Sorted by: year_pub author

© 2024 Montalvan-Sanchez EE et al. *JAMA Network Open.*

**eFigure 20. ADR (Adenomas Detection Rate) FIT programs by FIT cut-off. Leave-one-out analysis.**

| Study | Number of Adenomas | Total | Prevalence with 95% CI | Weight (%) |
|---|---|---|---|---|
| **50 ng/ml or Less** | | | | |
| Teixeira et al. (Brazil, 2017) | 43 | 73 | 58.90 [ 47.37, 69.98] | 9.88 |
| Fernandez et al. (Argentina, 2021) | 5 | 36 | 13.89 [ 4.18, 27.40] | 8.58 |
| Ruiz et al. (Argentina, 2021) | 121 | 553 | 21.88 [ 18.53, 25.43] | 11.36 |
| Heterogeneity: $\tau^2 = 0.24$, $I^2 = 95.82\%$, $H^2 = 23.90$ | | | 30.42 [ 8.30, 58.71] | |
| Test of $\theta_i = \theta_j$: Q(2) = 41.33, p = 0.00 | | | | |
| **100 ng/ml** | | | | |
| Fenocchi et al. (Uruguay, 2006) | 330 | 879 | 37.54 [ 34.37, 40.77] | 11.46 |
| Lopez-Kostner et al. (Chile, 2012) | 136 | 279 | 48.75 [ 42.89, 54.62] | 11.11 |
| Galvez-Rios et al. (Mexico, 2020) | 11 | 40 | 27.50 [ 14.61, 42.51] | 8.80 |
| Okada et al. (Chile, 2020) | 1,396 | 2,980 | 46.85 [ 45.06, 48.64] | 11.58 |
| Remes-Troche et al. (Mexico, 2020) | 6 | 20 | 30.00 [ 11.56, 52.18] | 7.12 |
| Heterogeneity: $\tau^2 = 0.02$, $I^2 = 90.46\%$, $H^2 = 10.48$ | | | 40.82 [ 33.54, 48.30] | |
| Test of $\theta_i = \theta_j$: Q(4) = 32.53, p = 0.00 | | | | |
| **Not reported** | | | | |
| Braga et al. (Brazil, 2017) | 12 | 35 | 34.29 [ 19.32, 50.94] | 8.51 |
| Alfaro-Seguro et al. (Costa Rica, 2020) | 852 | 3,761 | 22.65 [ 21.33, 24.01] | 11.59 |
| Heterogeneity: $\tau^2 = 0.02$, $I^2 = 60.37\%$, $H^2 = 2.52$ | | | 25.83 [ 15.86, 37.22] | |
| Test of $\theta_i = \theta_j$: Q(1) = 2.52, p = 0.11 | | | | |
| **Overall** | | | 34.16 [ 25.54, 43.32] | |
| Heterogeneity: $\tau^2 = 0.08$, $I^2 = 98.04\%$, $H^2 = 51.14$ | | | | |
| Test of $\theta_i = \theta_j$: Q(9) = 540.60, p = 0.00 | | | | |
| Test of group differences: $Q_b(2) = 4.92$, p = 0.09 | | | | |

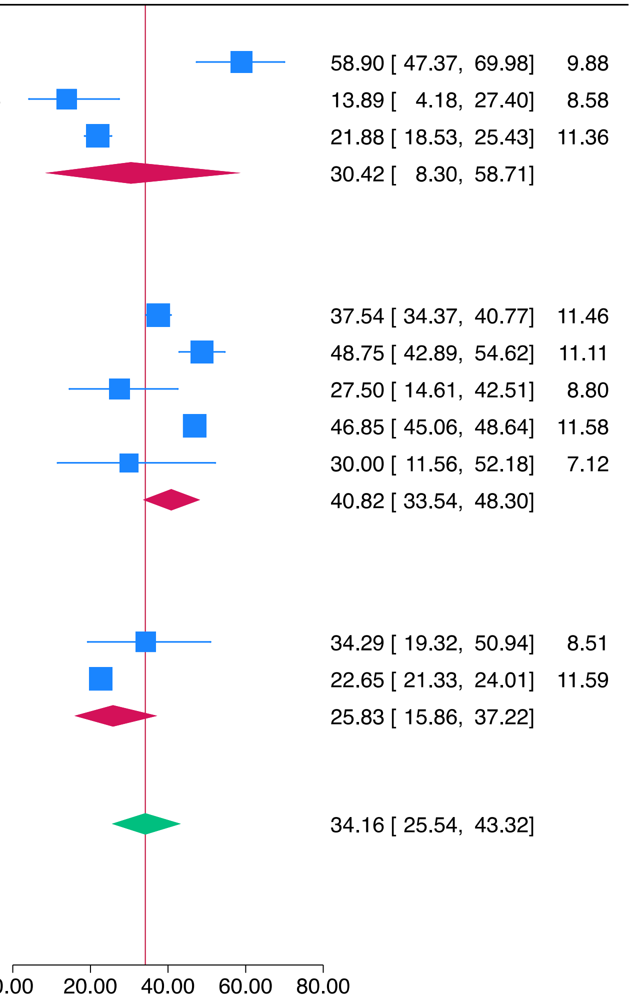


Random-effects REML model
Sorted by: year_pub author

*Leave-one-out analysis excluding Guimares et. al (2021) use of chromoendoscopy and Manzano-Robleda et. al (2020) use of endocuff.

© 2024 Montalvan-Sanchez EE et al. *JAMA Network Open.*

**eFigure 21. CRC (Adenocarcinoma Detection Rate) FIT programs. Leave-one-out analysis.**

| Study | Number of CRC's | Total | Prevalence with 95% CI | Weight (%) |
|---|---|---|---|---|
| **50 ng/ml or Less** | | | | |
| Teixeira et al. (Brazil, 2017) | 11 | 73 | 15.07 [ 7.66, 24.30] | 9.43 |
| Fernandez et al. (Argentina, 2021) | 2 | 36 | 5.56 [ 0.10, 16.02] | 7.37 |
| Ruiz et al. (Argentina, 2021) | 18 | 553 | 3.25 [ 1.92, 4.91] | 12.40 |
| Heterogeneity: $\tau^2 = 0.05$, $I^2 = 81.59\%$, $H^2 = 5.43$ | | | 6.87 [ 1.31, 15.70] | |
| Test of $\theta_i = \theta_j$: Q(2) = 13.09, p = 0.00 | | | | |
| **100 ng/ml** | | | | |
| Fenocchi et al. (Uruguay, 2006) | 101 | 879 | 11.49 [ 9.46, 13.69] | 12.63 |
| Lopez-Kostner et al. (Chile, 2012) | 3 | 279 | 1.08 [ 0.13, 2.71] | 11.84 |
| Galvez-Rios et al. (Mexico, 2020) | 1 | 40 | 2.50 [ 1.95, 10.42] | 7.70 |
| Okada et al. (Chile, 2020) | 180 | 2,980 | 6.04 [ 5.21, 6.92] | 12.91 |
| Remes-Troche et al. (Mexico, 2020) | 0 | 20 | 0.00 [ 0.01, 8.43] | 5.50 |
| Heterogeneity: $\tau^2 = 0.04$, $I^2 = 94.59\%$, $H^2 = 18.48$ | | | 3.92 [ 0.79, 8.80] | |
| Test of $\theta_i = \theta_j$: Q(4) = 55.06, p = 0.00 | | | | |
| **Not reported** | | | | |
| Braga et al. (Brazil, 2017) | 3 | 35 | 8.57 [ 1.14, 20.59] | 7.28 |
| Alfaro-Seguro et al. (Costa Rica, 2020) | 50 | 3,761 | 1.33 [ 0.99, 1.72] | 12.93 |
| Heterogeneity: $\tau^2 = 0.07$, $I^2 = 82.27\%$, $H^2 = 5.64$ | | | 3.27 [ 0.00, 14.09] | |
| Test of $\theta_i = \theta_j$: Q(1) = 5.64, p = 0.02 | | | | |
| **Overall** | | | 4.46 [ 1.91, 7.83] | |
| Heterogeneity: $\tau^2 = 0.04$, $I^2 = 95.88\%$, $H^2 = 24.26$ | | | | |
| Test of $\theta_i = \theta_j$: Q(9) = 232.92, p = 0.00 | | | | |
| Test of group differences: $Q_b(2) = 0.62$, p = 0.73 | | | | |

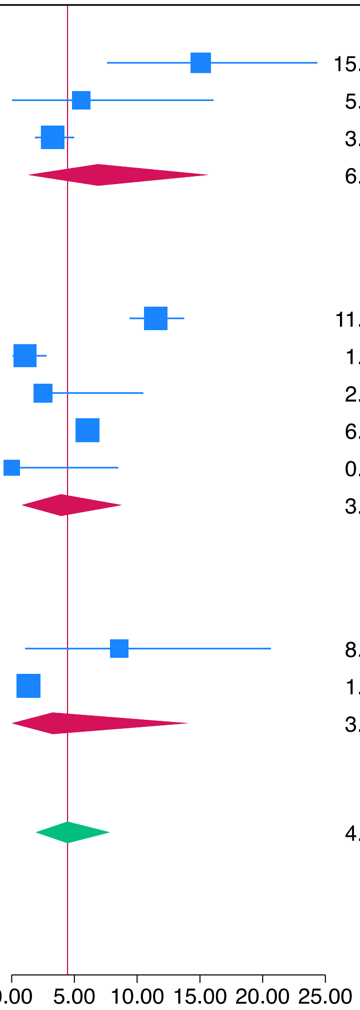


Random-effects REML model
Sorted by: year_pub author

*Leave-one-out analysis excluding Guimares et. al (2021) use of chromoendoscopy and Manzano-Robleda et. al (2020) use of endocuff.

© 2024 Montalvan-Sanchez EE et al. *JAMA Network Open.*

**eFigure 22. ADR (Adenomas Detection Rate) FIT programs. Leave-one-out analysis.**

| Omitted study | ADR with 95% CI | p-value |
|---|---|---|
| Fenocchi et al. (Uruguay, 2006) | 39.15 [ 28.45, 50.38] | 0.000 |
| Lopez-Kostner et al. (Chile, 2012) | 38.06 [ 27.61, 49.08] | 0.000 |
| Braga et al. (Brazil, 2017) | 39.39 [ 28.90, 50.38] | 0.000 |
| Teixeira et al. (Brazil, 2017) | 37.29 [ 27.41, 47.73] | 0.000 |
| Alfaro-Seguro et al. (Costa Rica, 2020) | 40.86 [ 30.72, 51.40] | 0.000 |
| Galvez-Rios et al. (Mexico, 2020) | 40.00 [ 29.61, 50.85] | 0.000 |
| Manzano-Robleda et al. (Mexico, 2020) | 37.10 [ 27.41, 47.32] | 0.000 |
| Okada et al. (Chile, 2020) | 38.22 [ 27.68, 49.33] | 0.000 |
| Remes-Troche et al. (Mexico, 2020) | 39.65 [ 29.30, 50.47] | 0.000 |
| Fernandez et al. (Argentina, 2021) | 41.32 [ 31.71, 51.26] | 0.000 |
| Guimaraes et al. (Brazil, 2021) | 36.47 [ 27.34, 46.11] | 0.000 |
| Ruiz et al. (Argentina, 2021) | 40.93 [ 30.85, 51.40] | 0.000 |

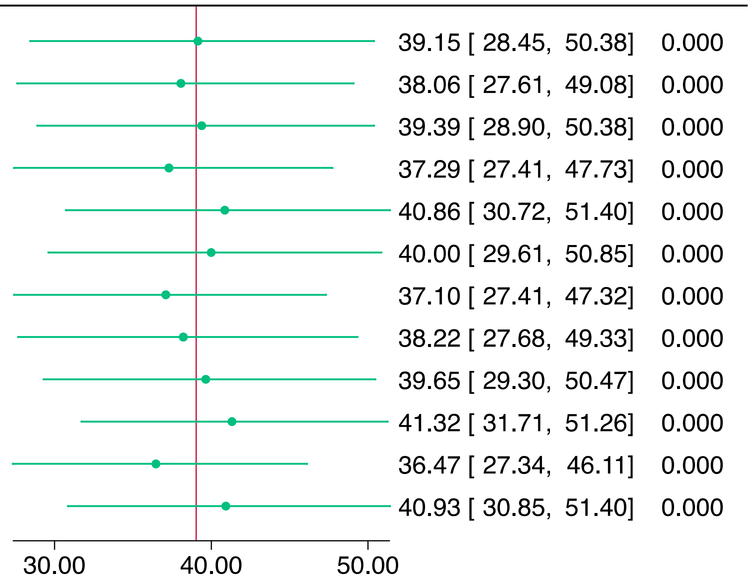


Random-effects REML model
Sorted by: year_pub author

For each study, the displayed effect size corresponds to an overall effect size computed from a meta-analysis excluding that study. The leave-one-out forest plot also displays a vertical line at the overall effect size based on the complete set of studies (with no omission) to help detect influential studies. The omission of study 4 (Teixeira et al 2017), 5 (Alfaro-Seguro et al. 2020), 7 (Manzano-Robleda et al. 2020) 10 (Fernandez et al. 2021), 11 (Guimares et al. 2021) or 12 (Ruiz et. al 2021) have the largest influence (when compared with other studies) on the estimation of the overall effect size.

© 2024 Montalvan-Sanchez EE et al. *JAMA Network Open.*

## eFigure 23. AADR (Advanced Adenomas Detection Rate) FIT programs. Leave-one-out analysis.

| Omitted study | AADR with 95% CI | p-value |
|---|---|---|
| Fenocchi et al. (Uruguay, 2006) | 11.97 [ 6.22, 19.18] | 0.000 |
| Lopez-Kostner et al. (Chile, 2012) | 14.14 [ 7.57, 22.26] | 0.000 |
| Fenocchi et al. (Uruguay, 2015) | 13.95 [ 7.34, 22.16] | 0.000 |
| Teixeira et al. (Brazil, 2017) | 12.33 [ 6.40, 19.78] | 0.000 |
| Alfaro-Seguro et al. (Costa Rica, 2020) | 15.31 [ 9.43, 22.27] | 0.000 |
| Galvez-Rios et al. (Mexico, 2020) | 13.97 [ 7.50, 21.97] | 0.000 |
| Manzano-Robleda et al. (Mexico, 2020) | 11.67 [ 6.41, 18.18] | 0.000 |
| Fernandez et al. (Argentina, 2021) | 13.86 [ 7.41, 21.87] | 0.000 |
| Guimaraes et al. (Brazil, 2021) | 12.45 [ 6.32, 20.18] | 0.000 |
| Ruiz et al. (Argentina, 2021) | 14.07 [ 7.48, 22.24] | 0.000 |

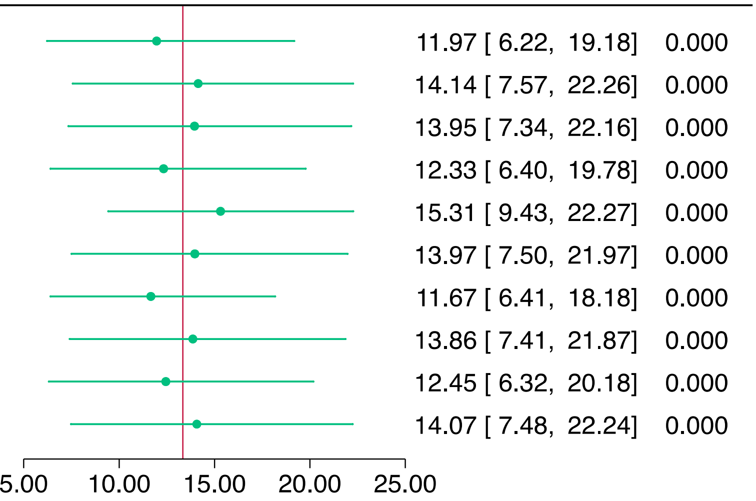


Random-effects REML model
Sorted by: year_pub author

For each study, the displayed effect size corresponds to an overall effect size computed from a meta-analysis excluding that study. The leave-one-out forest plot also displays a vertical line at the overall effect size based on the complete set of studies (with no omission) to help detect influential studies. The omission of study 1 (Fenocchi et al. 2006), 5 (Alfaro-Seguro et al. 2020), and 7 (Manzano-Robleda et al, 2020) have the largest influence (when compared with other studies) on the estimation of the overall effect size.

© 2024 Montalvan-Sanchez EE et al. *JAMA Network Open.*

**eFigure 24. CRC (Adenocarcinoma Detection Rate) FIT programs. Leave-one-out analysis.**

| Omitted study | CRC-DR with 95% CI | p-value |
|---|---|---|
| Fenocchi et al. (Uruguay, 2006) | 4.73 [ 2.61, 7.35] | 0.000 |
| Lopez-Kostner et al. (Chile, 2012) | 5.54 [ 3.01, 8.67] | 0.000 |
| Braga et al. (Brazil, 2017) | 5.28 [ 2.96, 8.15] | 0.000 |
| Teixeira et al. (Brazil, 2017) | 4.79 [ 2.73, 7.32] | 0.000 |
| Alfaro-Seguro et al. (Costa Rica, 2020) | 6.16 [ 3.98, 8.72] | 0.000 |
| Galvez-Rios et al. (Mexico, 2020) | 5.64 [ 3.21, 8.62] | 0.000 |
| Manzano-Robleda et al. (Mexico, 2020) | 5.01 [ 2.79, 7.75] | 0.000 |
| Okada et al. (Chile, 2020) | 5.38 [ 2.86, 8.54] | 0.000 |
| Remes-Troche et al. (Mexico, 2020) | 5.82 [ 3.44, 8.71] | 0.000 |
| Fernandez et al. (Argentina, 2021) | 5.43 [ 3.05, 8.38] | 0.000 |
| Guimaraes et al. (Brazil, 2021) | 5.49 [ 2.95, 8.65] | 0.000 |
| Ruiz et al. (Argentina, 2021) | 5.75 [ 3.23, 8.84] | 0.000 |

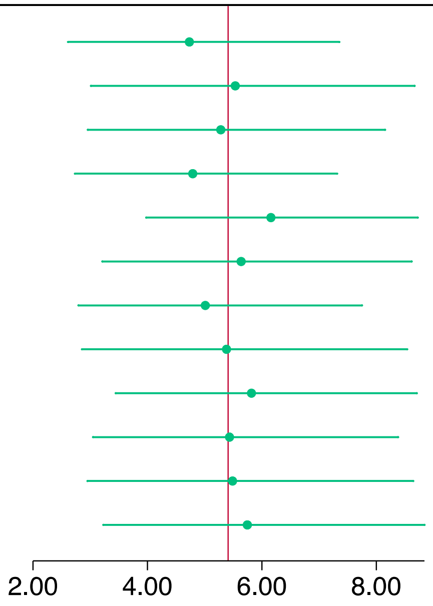


Random-effects REML model
Sorted by: year_pub author

For each study, the displayed effect size corresponds to an overall effect size computed from a meta-analysis excluding that study. The leave-one-out forest plot also displays a vertical line at the overall effect size based on the complete set of studies (with no omission) to help detect influential studies. The omission of study 1 (Fenocchi et al. 2015) and 4 (Teixeira et al. 2017) have the largest negative effect on pooled CRC detection, while studies 5 (Alfaro-Seguro et al. 2020), and 9 (Remes-Troche et al. et al 2020) have the largest negative effect.

© 2024 Montalvan-Sanchez EE et al. *JAMA Network Open.*
