# Supplementary material for: Colorectal Cancer Screening Programs in Latin America: A Systematic Review and Meta-Analysis
Source: JAMA Netw Open. 2024 Feb 1;7(2):e2354256. doi: 10.1001/jamanetworkopen.2023.54256 (PMC10835514; doi:10.1001/jamanetworkopen.2023.54256)
Supplement: Supplement 2. — Data Sharing Statement [file jamanetwopen-e2354256-s002.pdf]

Data Sharing Statement

Montalvan-Sanchez. Colorectal Cancer Screening Programs in Latin America. *JAMA Netw Open*. Published February 01, 2024. doi:10.1001/jamanetworkopen.2023.54256

Data
Data available: No
